# Homogeneous copper-catalyzed aerobic oxidation of alcohols and aldehydes: mechanistic insights and recent advances

**DOI:** 10.3762/bjoc.22.107

**Published:** 2026-09-29

**Authors:** Hristo Petkov, Svilen P Simeonov

**Affiliations:** 1 Institute of Organic Chemistry with Centre of Phytochemistry, Bulgarian Academy of Sciences, Acad. G. Bonchev St., Bl. 9, 1113 Sofia, Bulgaria; 2 Research Institute for Medicines (iMed.ULisboa), Faculty of Pharmacy, Universidade de Lisboa, 1649-003 Lisbon, Portugal

**Keywords:** aerobic oxidation, alcohol oxidation, aldehyde oxidation, copper catalysis, mechanism

## Abstract

Copper-catalyzed aerobic oxidation of alcohols and aldehydes has become a key transformation for the sustainable synthesis of carbonyl compounds, combining high efficiency with the use of molecular oxygen as a benign terminal oxidant. Owing to its earth-abundance, low cost, low toxicity, and versatile redox properties, copper offers a compelling alternative to traditional noble metal catalysts and stoichiometric oxidation methods. This review highlights the evolution of copper-catalyzed aerobic oxidation of alcohols, emphasizing two distinct mechanistic regimes: nitroxyl radical-mediated systems and nitroxyl-free pathways, in which copper–ligand complexes directly promote dioxygen activation and substrate oxidation. Methods for conversion of aldehydes to carboxylic acids were also discussed. Apart from historical overview of the considered protocols, recent advances in the field were deliberated. Despite significant progress, the development of simpler, mediator-free catalytic systems based on readily available reagents remains a central challenge and a promising direction for future research.

## Introduction

Carbonyl compounds, particularly aldehydes and ketones, are extremely valued commodities and building blocks in the pharmaceutical and chemical industries [1–9]. Beyond their use in traditional thermochemical synthesis, they have also found applications in modern fields like photochemistry [10] and electrochemistry [11]. These chemicals of higher oxidation state and importance are most often prepared via oxidation of alcohols [12]. Conventional oxidation methods in organic synthesis often use stoichiometric oxidants such as chromium reagents [13], permanganates [14], or hypervalent iodine reagents [15], as well as classical protocols such as Swern [16], Corey–Kim [17], Parikh–Doering [18] and Pinnick [19] oxidations. While these traditional approaches have proved historically essential and remain in use today, they typically generate significant amounts of waste and are no longer favored due to sustainability issues [20–21]. In the pursuit of greener protocols, strategies such as catalytic Oppenauer-type [22] and organocatalytic solvent-free [23] oxidations have also been developed. Nevertheless, systems utilizing abundant first-row transition metals remain particularly attractive, among which copper plays a central role. Beyond copper chemistry, significant research efforts have also been devoted to the development of iron-catalyzed oxidations [24], yielding several robust, sustainable and widely recognized protocols [25–28].

Copper-catalyzed oxidation reactions offer a compelling and environmentally benign alternative to traditional methods, especially when molecular oxygen is employed as the terminal oxidant [29]. Copper has gained prominence due to its earth-abundance, low cost, and rich redox chemistry, and thus offers an appealing option from both economic and environmental perspectives [29–30]. In addition to these advantages, when compared to other transition metals, copper exhibits a remarkably lower toxicity profile. According to the ICH Q3D (R2) guidelines on elemental impurities [31], copper features a high oral permitted daily exposure (3400 µg/day), allowing residual concentrations of up to 340 ppm in final products, whereas much stricter limits apply to metals such as gold (32 ppm), nickel (22 ppm), palladium (10 ppm) and cobalt (5 ppm). Since traces of toxic metal impurities are often difficult to remove from the products, this regulatory advantage of copper significantly minimizes the costs and complexity associated with post-synthetic purification in the agrochemical and pharmaceutical industries.

In recent decades, copper catalysis has evolved considerably, with the development of ligand-enabled variants that have opened new avenues for carbon–carbon and carbon–heteroatom-bond formation [32–34], as well as a broad range of oxidative transformations [35–37].

Among others, copper-catalyzed oxidation of alcohols to aldehydes or ketones, and of aldehydes to carboxylic acids, has been of central focus over the years [21,29]. These reactions are of fundamental importance for both laboratory and industrial chemistry, as they provide straightforward access to valuable chemicals. While several reviews have addressed related topics, many are now outdated or treat these transformations within broader contexts [21,29,36,38–43]. To address the lack of a focused and up-to-date overview specifically dedicated to these substrate classes, this review will discuss their relevant copper-catalyzed aerobic oxidation reactions.

**Scheme 1:**
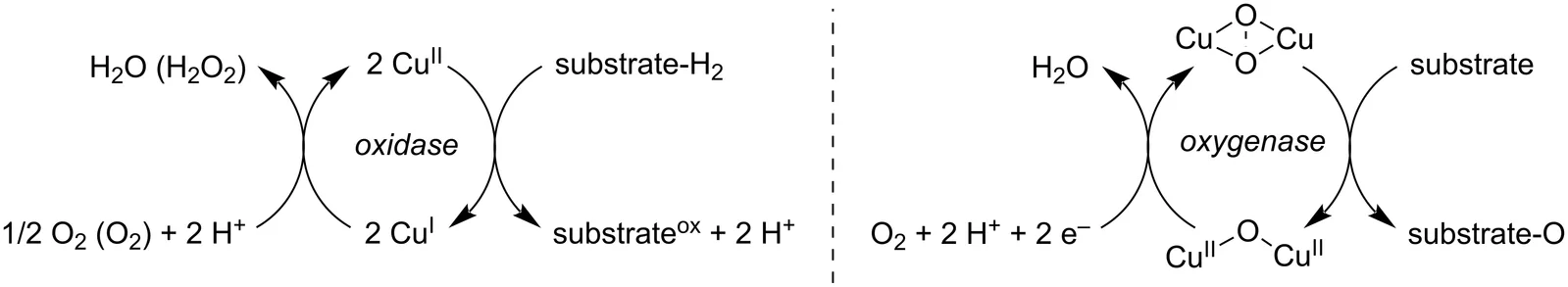
Oxidase- and oxygenase-like copper-based oxidation reactions.

The use of oxygen is particularly appealing from a sustainability point of view, as it is inexpensive, readily available, and produces water as the only stoichiometric by-product in most instances [44]. These important features have driven extensive research into natural aerobic pathways and the creation of novel synthetic methodologies based on them. In all of these processes, oxygen functions either as an oxidant reduced to water (oxidase-like activity), a reactant embedded into the final product (oxygenase-like activity), or fulfills both roles simultaneously. In copper-based oxidations the oxidase catalytic cycle simply utilizes O_2_ as a two-electron or two-proton acceptor in the catalytic oxidation of organic molecules. In the reaction, oxygen atoms are eventually reduced to either H_2_O or H_2_O_2_. Thus the transfer of O atoms to the substrate is not observed. In the oxygenase catalytic cycle, the oxidation of substrate involves O transfer from O_2_, often through a high-valent metal oxyl intermediate, while the other O atom is reduced to H_2_O (Scheme 1) [45–46].

**Scheme 2:**
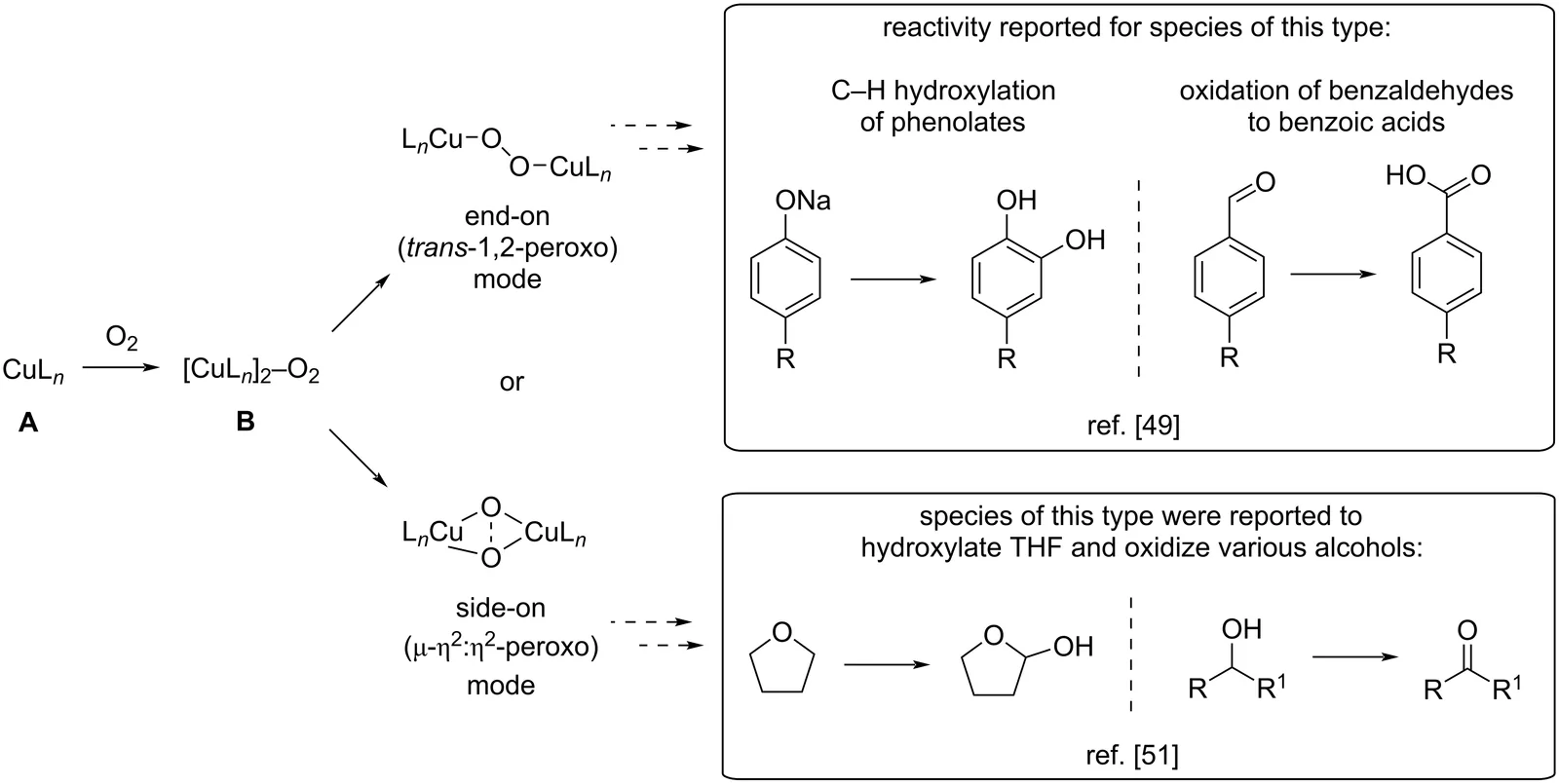
Formation and coordination modes (end-on vs side-on) of dioxygen–dicopper complexes and their synthetic applications in oxidation reactions.

Dioxygen–dicopper complexes have been extensively investigated as key species in copper-mediated oxidation reactions [47]. Mechanistically, a Cu(I) complex **A** reacts with O_2_ to generate a Cu_2_–O_2_ species **B**. The O_2_-derived unit may adopt different coordination geometries depending on the ligand environment, including end-on (μ-1,2-) [48–49] or side-on (μ-η^2^:η^2^) [50–51]. Although several Cu/O₂ species can be formed during dioxygen activation by copper, and some of them can interconvert [52–54], two of the most well-studied Cu_2_-O_2_ complexes, bearing *trans*-1,2-peroxo or (μ-η^2^:η^2^-peroxo)dicopper cores, have been proposed as reactive intermediates that promote substrate oxidation (Scheme 2).

This review encompasses the important historical background and development of Cu-catalyzed aerobic oxidation of alcohols and aldehydes, as well as the most recent reports on the topic. It also puts emphasis on the mechanistic details behind these processes. Cu-catalyzed aerobic oxidation reactions can be broadly categorized into two mechanistically distinct classes. The first type involves nitroxyl-radical-mediated systems, where a redox-active organic co-catalyst operates in synergy with copper to enable efficient oxidation. The second includes nitroxyl-radical-free systems, in which copper–ligand complexes mediate both substrate activation and dioxygen turnover in the absence of an external redox mediator. Despite sharing common catalytic elements, these two approaches differ in their operative mechanisms and catalytic roles of the individual components.

## Review

### Copper-catalyzed aerobic oxidation of alcohols to aldehydes and ketones

#### Nitroxyl radical-mediated Cu-catalyzed aerobic alcohol oxidation

The most widely used methods for aerobic alcohol oxidation rely on redox-active nitroxyl radicals in combination with Cu catalysts [42,46,55–57]. These radicals, especially when used together with the appropriate copper salt, have proven to be highly effective in selective and high-yielding aerobic alcohol oxidation reactions.

**Scheme 3:**
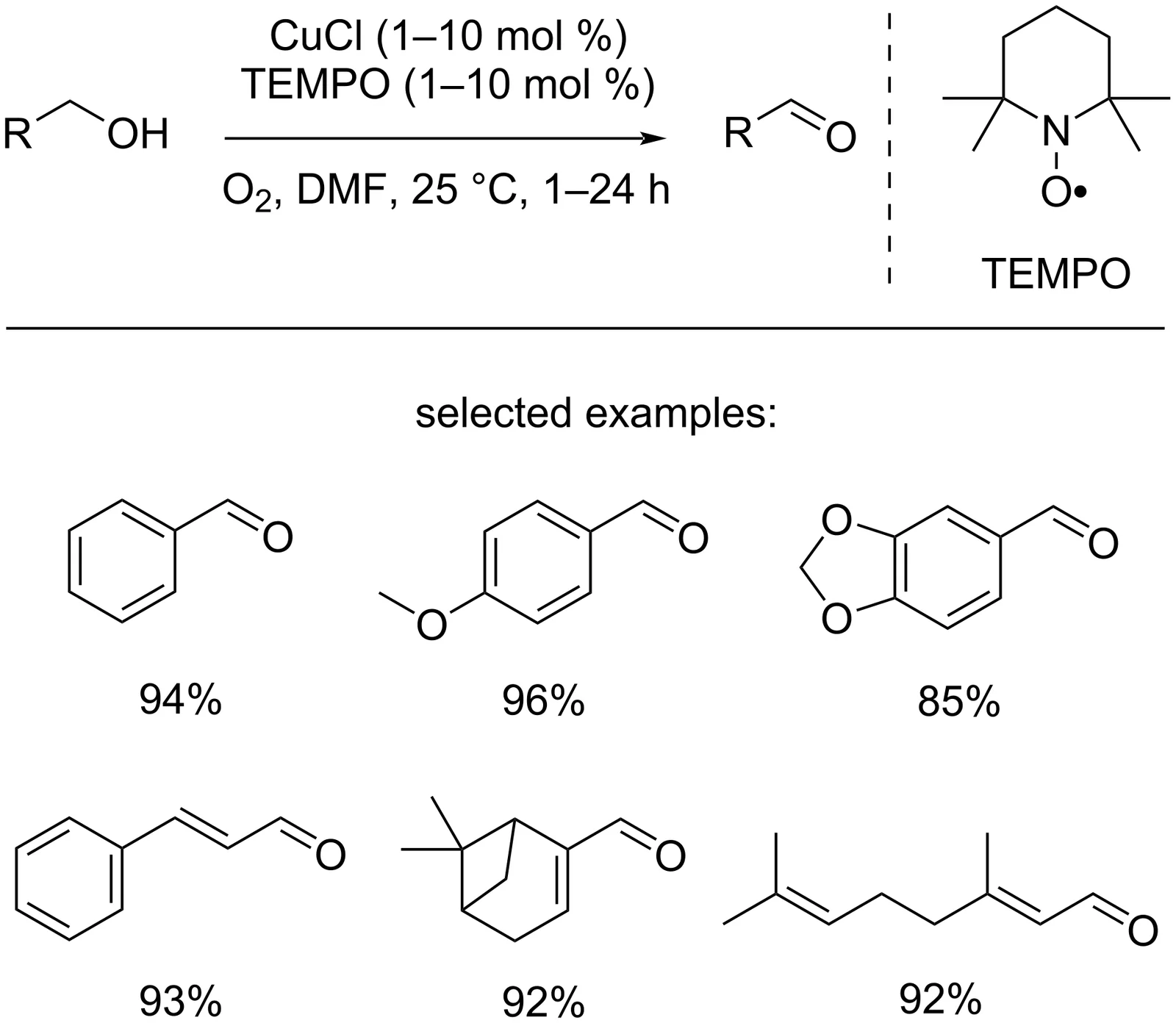
The first practical Cu-catalyzed aerobic oxidation of alcohols employing TEMPO.

In 1984, Semmelhack et al. reported the first practical Cu-catalyzed aerobic oxidation of alcohols to aldehydes [58], using Cu in combination with the stable 2,2,6,6-tetramethyl-1-piperidine-*N*-oxyl radical (TEMPO) in DMF as a solvent at room temperature (Scheme 3). Although this system operated without a ligand and generally achieved excellent yields of around 90% with no over-oxidation to acids, it was efficient only for primary activated (benzylic and allylic) alcohols.

**Scheme 4:**
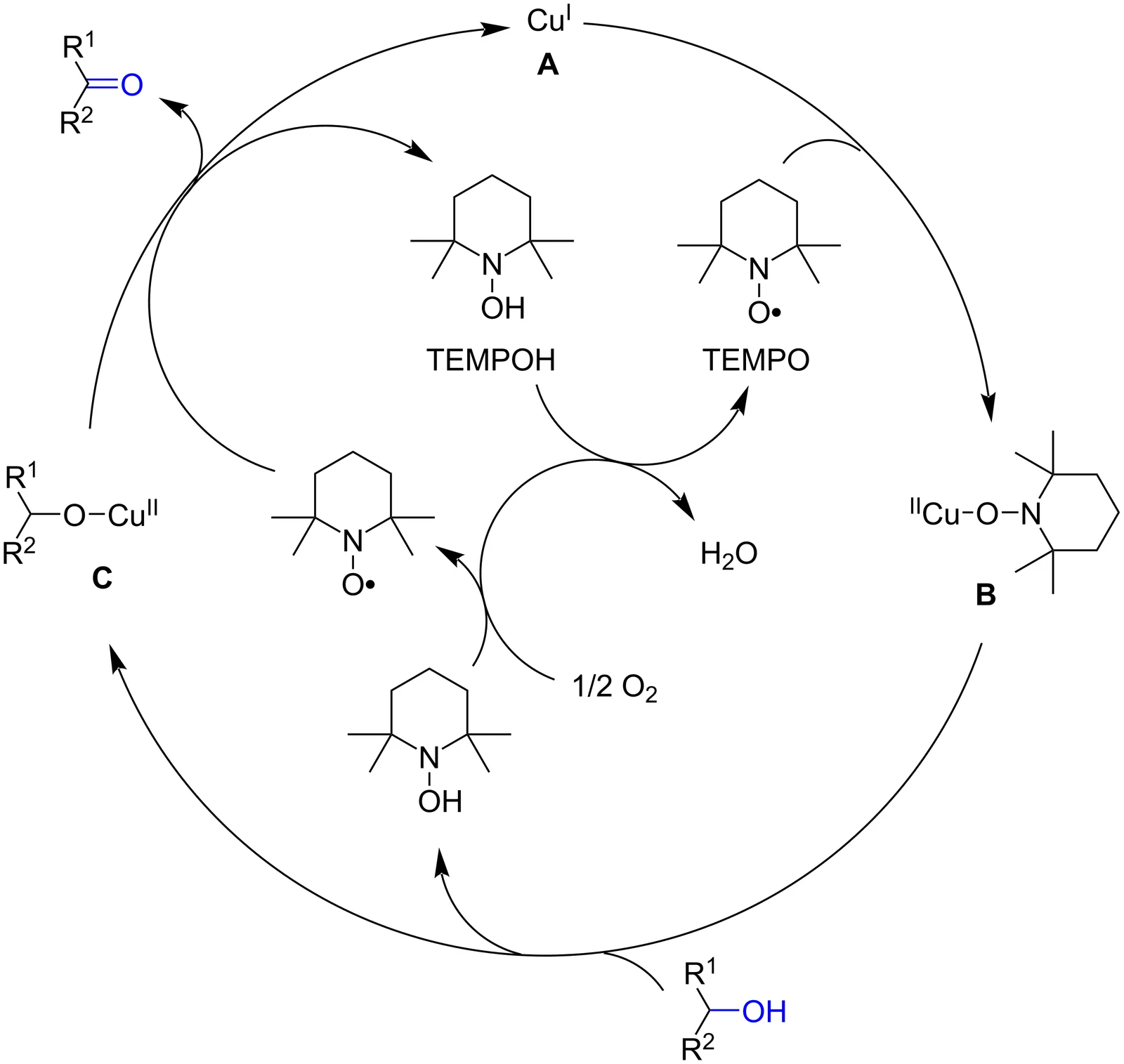
Revisited mechanism of the Cu/TEMPO-catalyzed aerobic oxidation of alcohols by Sheldon et al. [59].

While the pioneering work by Semmelhack and co-workers originally identified TEMPO as the alcohol oxidant, with CuCl merely serving as a co-catalyst that drives TEMPO recycling, follow-up mechanistic investigations by Sheldon et al. [59] fundamentally revised this picture. Their findings revealed that Cu actually functions as the direct, substrate-selective redox catalyst, whereas TEMPO serves as the mediator responsible for regenerating the active copper species (as illustrated in Scheme 4).

As shown in the proposed catalytic pathway (Scheme 4), the initial step involves the interaction of copper(I) species **A** with TEMPO to yield the corresponding intermediate **B**. Subsequent reaction of **B** with the alcohol substrate generates copper(II) alkoxide complex **C** alongside TEMPOH (the reduced form of TEMPO). Complex **C** then reacts with another equivalent of TEMPO, furnishing the carbonyl product, an additional molecule of TEMPOH, and regenerating the copper(I) catalyst **A**. To close the cycle, the formed TEMPOH is re-oxidized to TEMPO in the presence of molecular oxygen (Scheme 4) [59–60].

**Scheme 5:**
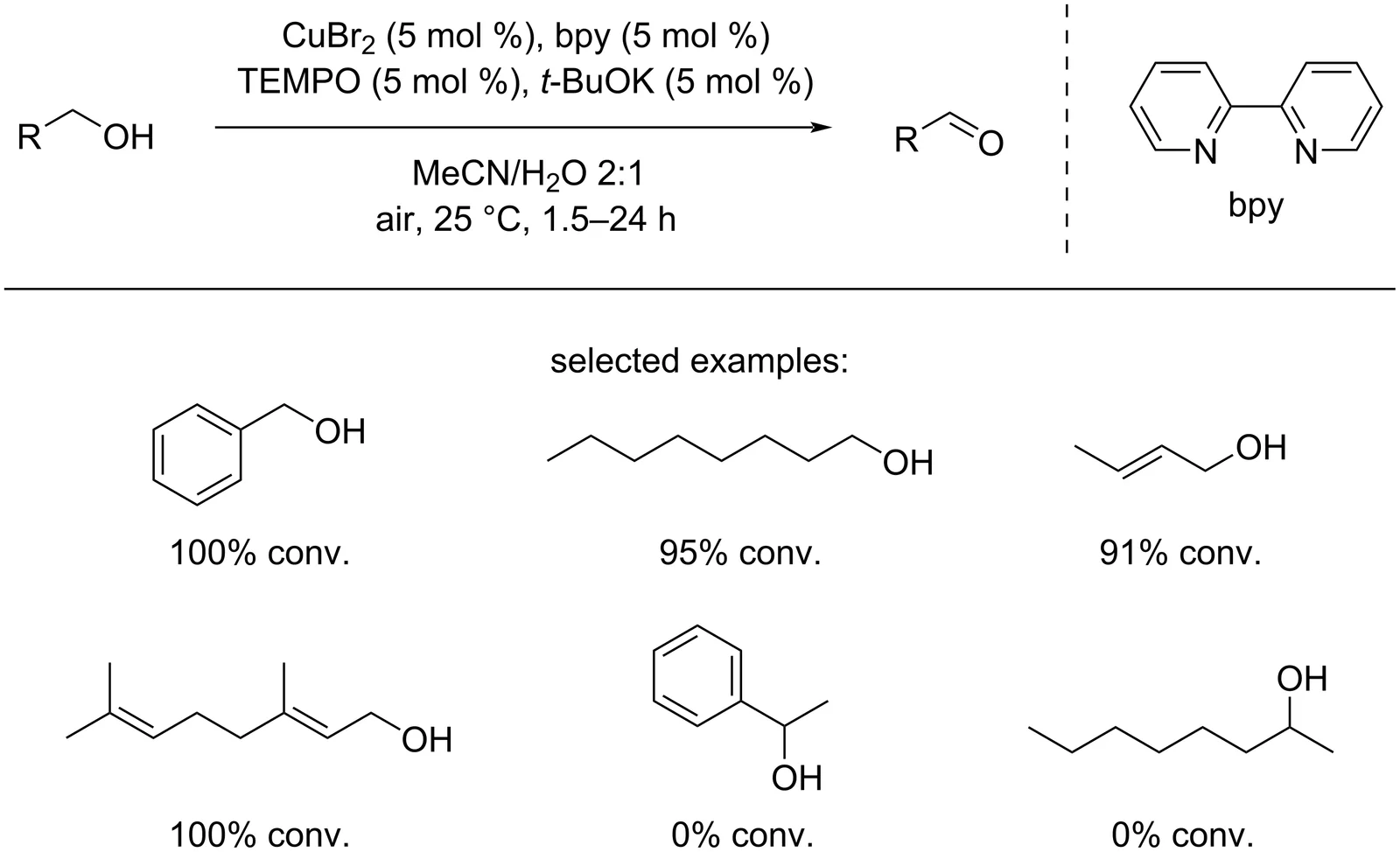
CuBr_2_/bpy/TEMPO-catalyzed oxidation of primary alcohols to aldehydes under air.

Furthermore, Sheldon and co-workers demonstrated that the combination of CuBr_2_ and TEMPO, together with 2,2′-bipyridine (bpy) as a ligand, efficiently promotes the oxidation of primary alcohols to aldehydes without over-oxidation to acids [61]. A major feature of this remarkably mild protocol is its ability to deliver excellent conversions under ambient air (rather than pure oxygen) at room temperature. The method also exhibited excellent chemoselectivity as only primary alcohols were converted, in contrast to secondary alcohols’ lack of reactivity (Scheme 5).

**Figure 1:**
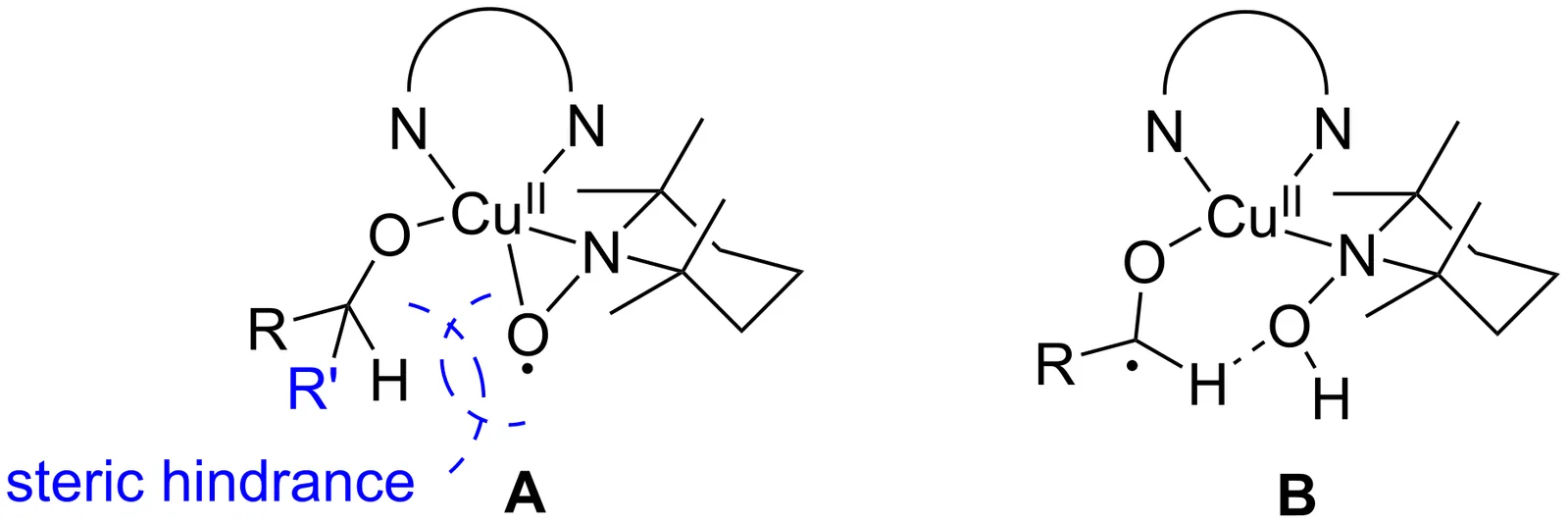
Possible explanation for the lack of reactivity of secondary alcohols proposed by Sheldon et al. [61].

The complete lack of reactivity observed for secondary alcohols was attributed to sterics [61]. The presence of an additional R′ group in the secondary alcohols impairs the formation of species **A**, whereas its absence in primary alcohols allows for the stabilizing effect of the second β-hydrogen which facilitates the formation of radical species **B** (Figure 1).

**Scheme 6:**
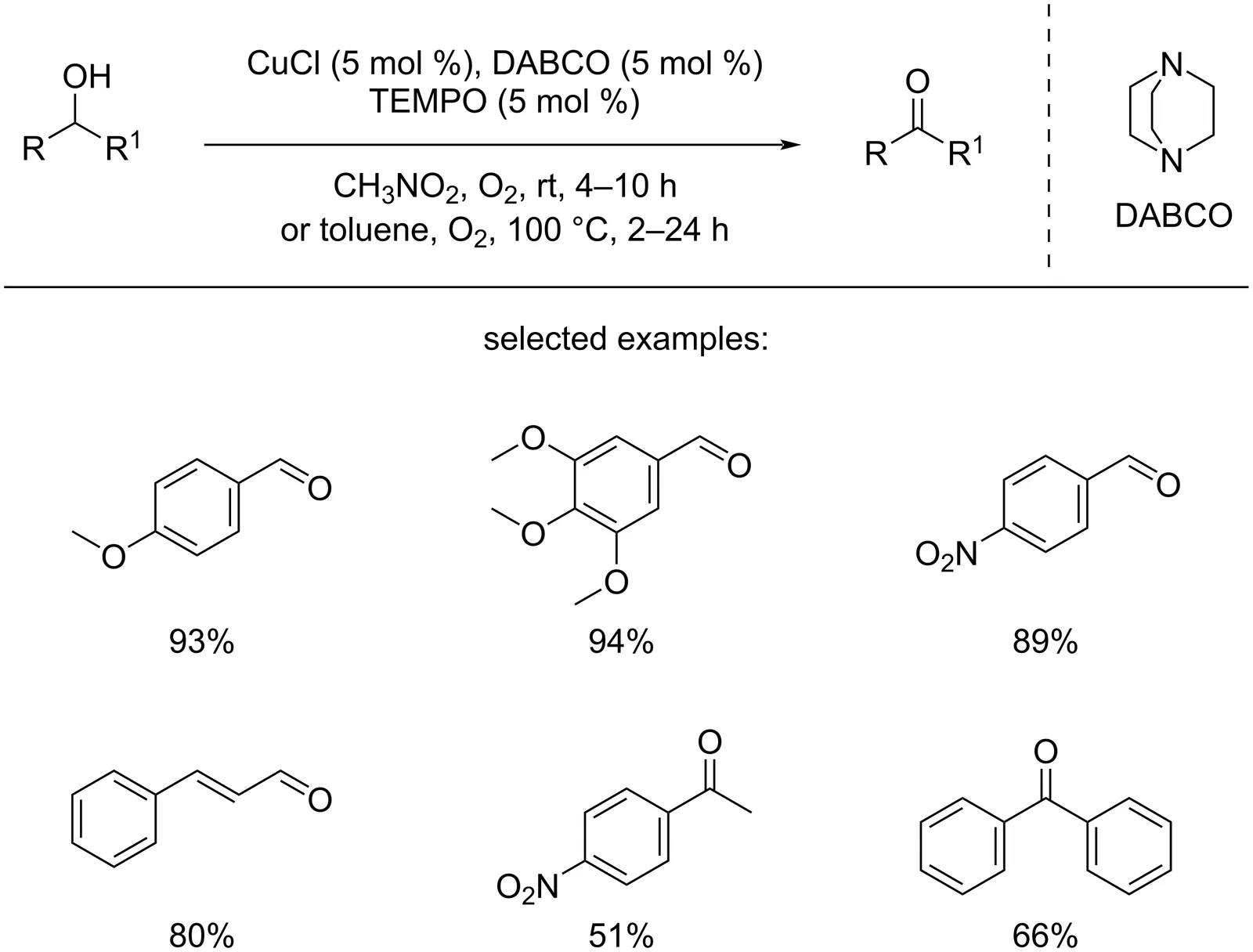
The CuCl/DABCO/TEMPO system for the aerobic oxidation of activated alcohols.

In 2007, the group of Sekar [62] reported a method that utilizes CuCl, TEMPO, and 1,4-diazabicyclo[2.2.2]octane (DABCO), all in 5 mol %. The latter is suggested by the authors to serve a twofold purpose: deprotonating the alcohol’s hydroxy group as a base and coordinating to CuCl as an N-donor ligand. Using nitromethane as a solvent under an oxygen atmosphere, various primary activated alcohols were smoothly transformed into the corresponding aldehydes at ambient conditions. Secondary alcohols, however, were found to be more challenging, requiring a high temperature (100 °C in toluene) and prolonged reaction time (Scheme 6). In addition, the catalytic system was proven insufficient for oxidizing aliphatic alcohols with satisfactory efficiency.

**Scheme 7:**
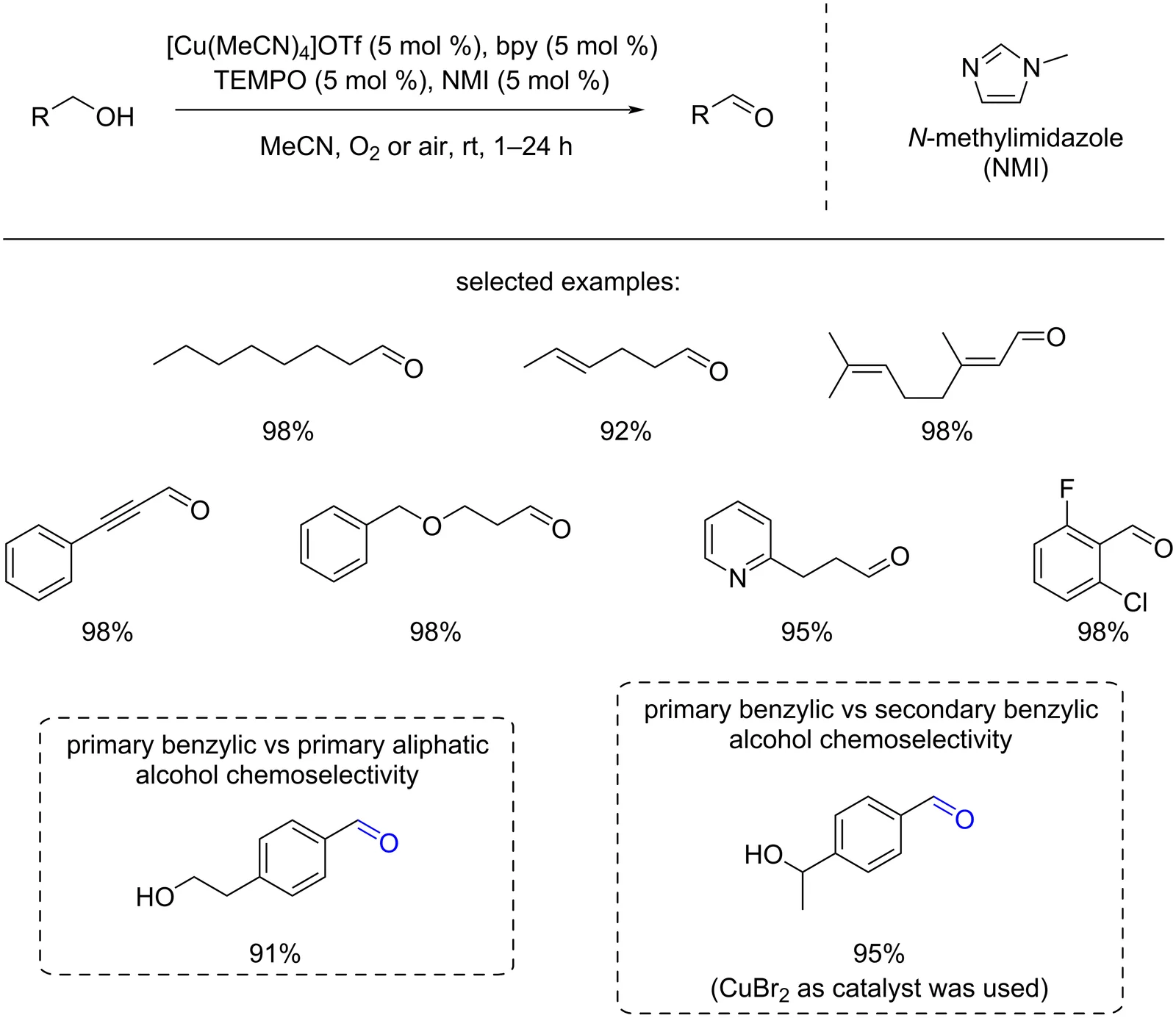
The Cu/bpy/TEMPO/NMI system for the chemoselective aerobic oxidation of primary alcohols.

In 2011, an extensive study conducted by Hoover and Stahl discovered a new copper-based catalytic system (Cu/bpy/TEMPO/NMI) which showed a broad scope, excellent functional group tolerance, high efficiency towards oxidation of aliphatic substrates, and exquisite selectivity for primary alcohols [63]. Moreover, taking advantage of these features, the authors achieved the selective oxidation of diols without the need of protecting groups (Scheme 7).

**Scheme 8:**
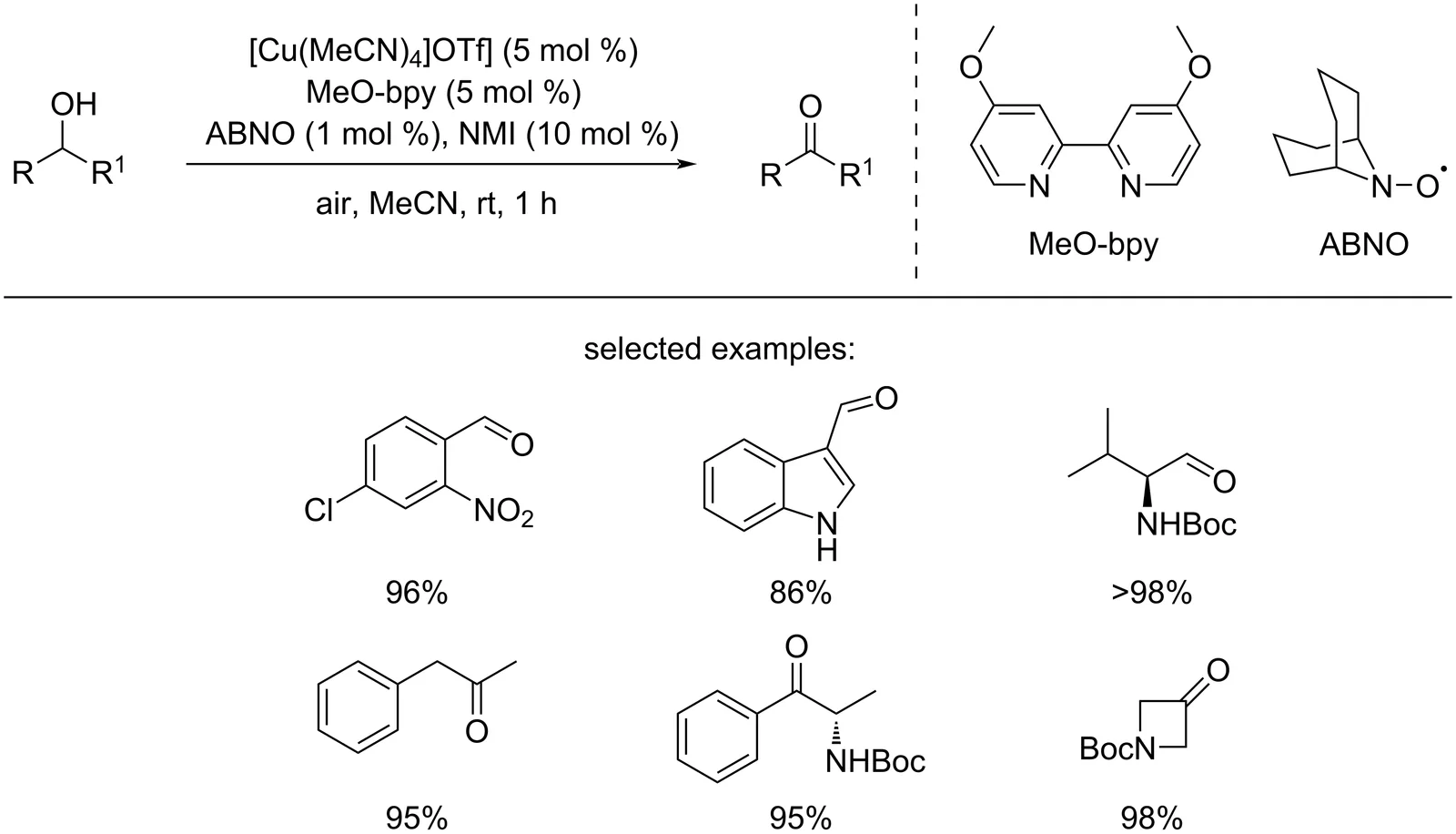
The Cu/ABNO catalytic system for the aerobic oxidation of both primary and secondary alcohols.

The insights from the method developed by Hoover and Stahl in 2011 [63] established a basis for overcoming the major limitation of the protocol, namely the non-reactivity of secondary alcohols. Although this method enables a highly chemoselective oxidation of primary alcohols over unprotected secondary ones, it comes at the expense of a narrower reaction scope. To overcome the limitations of their initial Cu/TEMPO system, in 2013, Steves and Stahl introduced a complementary catalytic system that employs the less sterically demanding nitroxyl radical 9-azabicyclo[3.3.1]nonane *N*-oxyl (ABNO) [64]. This refined approach exhibited exceptional catalytic activity across a broad range of substrates, smoothly converting primary as well as secondary alcohols, including benzylic, allylic, and aliphatic derivatives (Scheme 8). It is worth mentioning that although Stahl’s TEMPO- and ABNO-based catalytic systems are comprised of several components, it was later discovered that their acetonitrile solutions offer long-term stability (≥1 year) when refrigerated and can be used directly in reactions, thereby improving operational simplicity of the protocols. In addition, these solutions have been made commercially available [65].

**Scheme 9:**
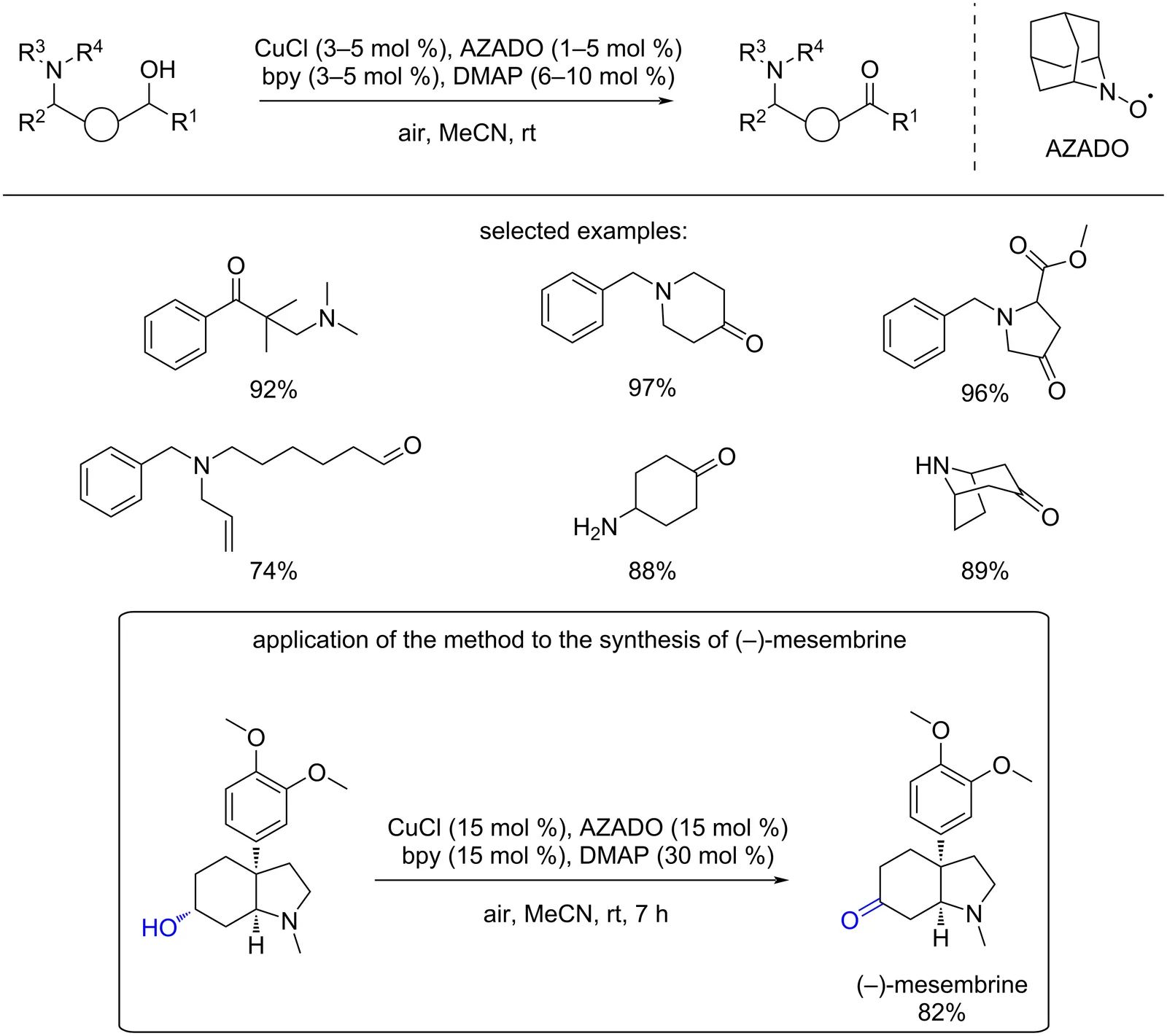
The Cu/AZADO catalytic system for the aerobic oxidation of amino alcohols.

While the protocol reported by Stahl and Steves [64] was tested on several amino-functionalized molecules, in 2014, the group of Iwabuchi [66] disclosed a Cu/AZADO system (AZADO = 2-azaadamantane *N*-oxyl) for the oxidation of a broader spectrum of amino alcohols (Scheme 9). The authors found that the AZADO radical was superior to TEMPO and ABNO, and with AZADO loadings of 1–5 mol % a series of primary and secondary alcohols bearing either protected or unprotected amine functionalities were oxidized in good to excellent yields. Moreover, the method was further applied to the synthesis of the natural product (−)-mesembrine (Scheme 9).

**Scheme 10:**
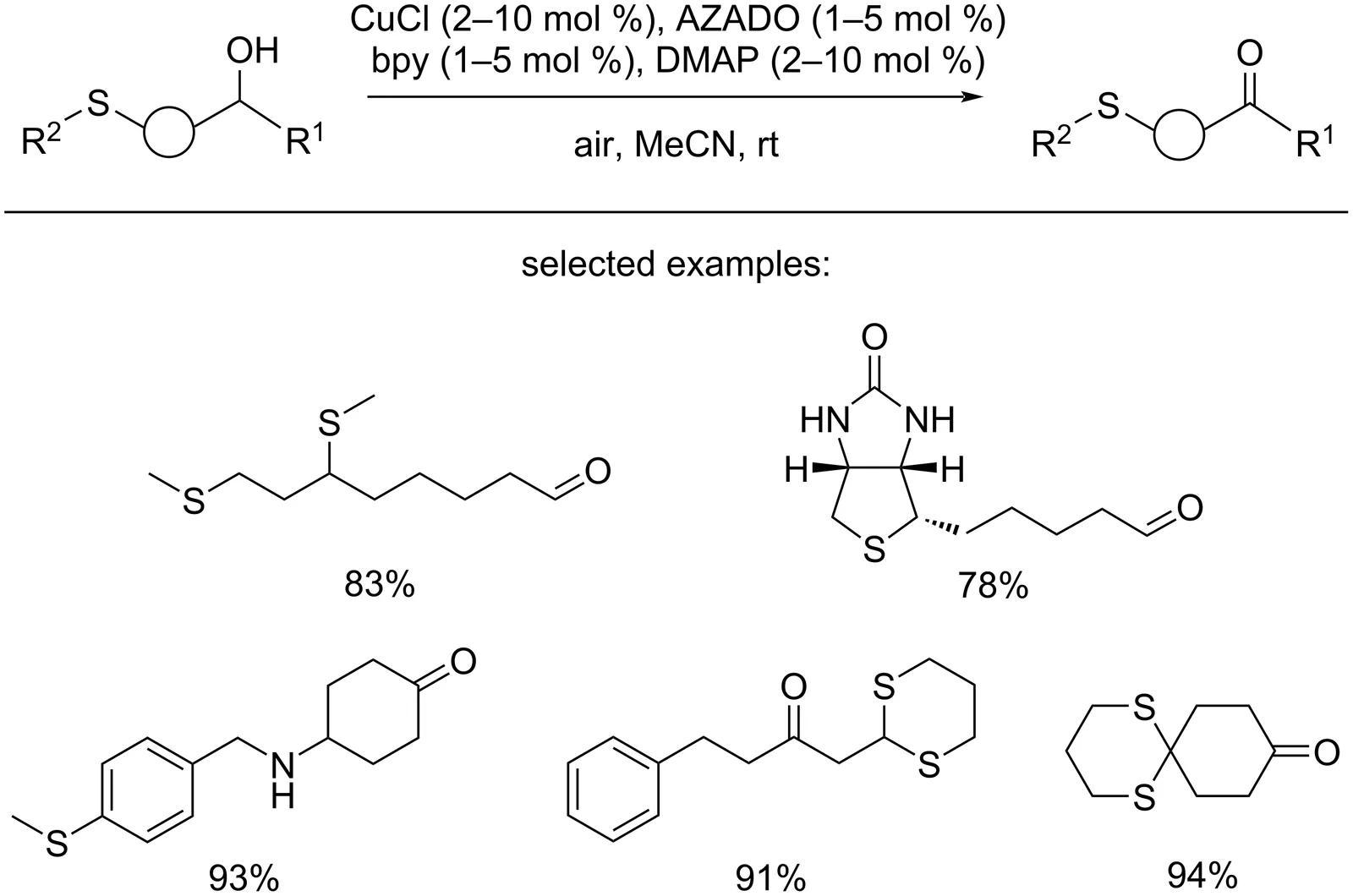
The Cu/AZADO catalytic system for the aerobic oxidation of sulfur-containing alcohols.

The group of Iwabuchi later extended the substrate scope of their catalytic system to oxidatively labile thioether-containing alcohols, including dithianes and sulfides [67]. Under optimized conditions, the chemoselective synthesis of the corresponding carbonyl products was achieved in good to high yields with preservation of the thioether groups (Scheme 10).

The success of the described above nitroxyl-mediated oxidation relies on the presence of both ligand and a base. The need of additional base comes from the understanding that it assists the catalytic cycle through deprotonation of the alcohol, thus enhancing the substrate coordination to the Cu center. Moreover, it stabilizes the reactive catalytic species and accelerates molecular oxygen activation [40–41,68]. In an attempt to eliminate the need of external base, new catalytic systems have emerged in recent years.

**Scheme 11:**
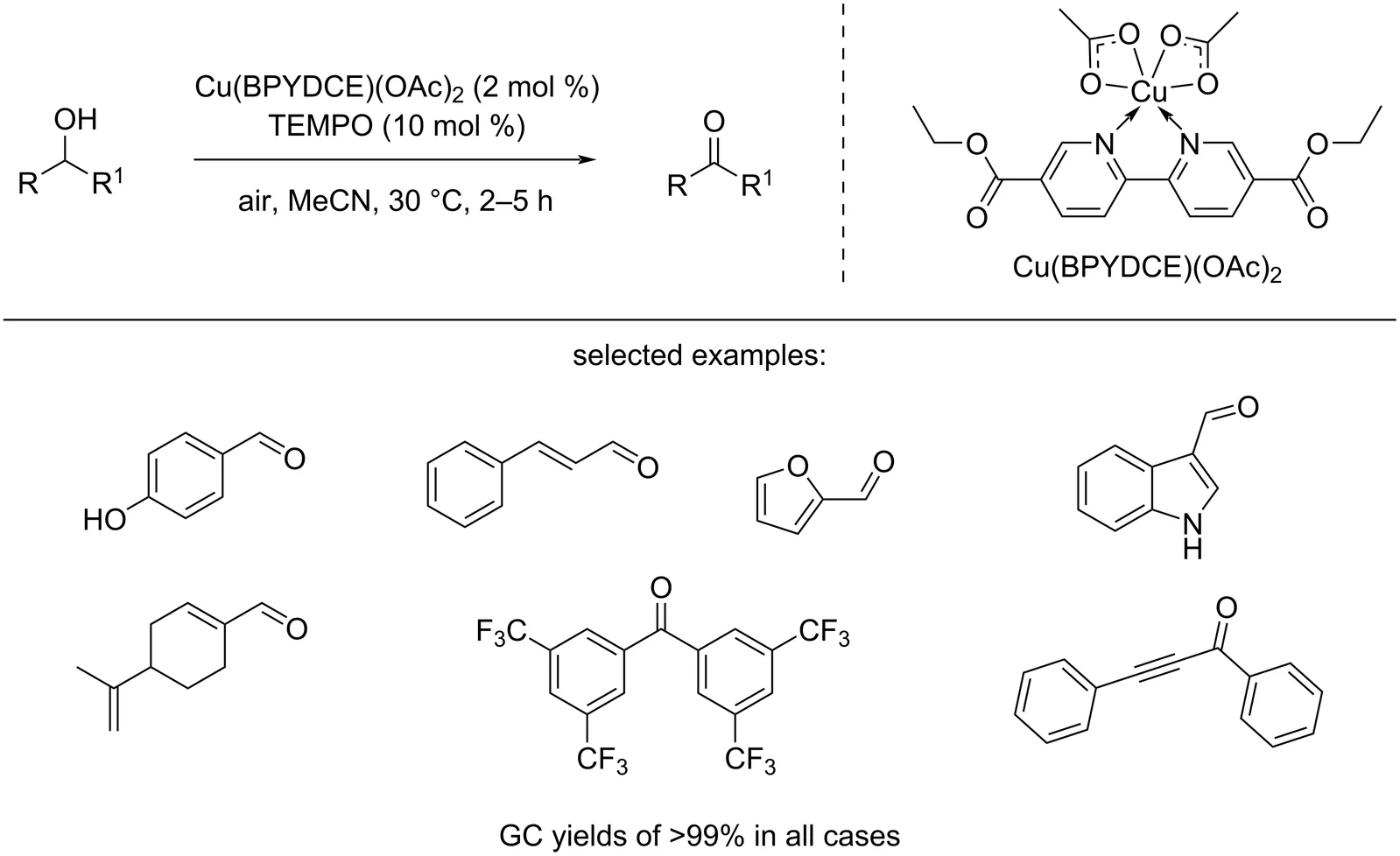
Cu(BPYDCE)(OAc)_2_/TEMPO-catalyzed external base-free aerobic oxidation of alcohols.

For instance, TEMPO was successfully employed as a co-catalyst in a combination with Cu(OAc)_2_/BPYDCDE complex (BPYDCDE = [2,2']bipyridinyl-5,5'-dicarboxylic acid diethyl ester) for the oxidation of both primary and secondary activated alcohols in the absence of exogenous base [69]. In all cases full conversions and analytical yields of >99% were achieved (based on GC analysis using *n*-decane as an internal standard), when the reactions were conducted under air at 30 °C in MeCN (Scheme 11).

**Scheme 12:**
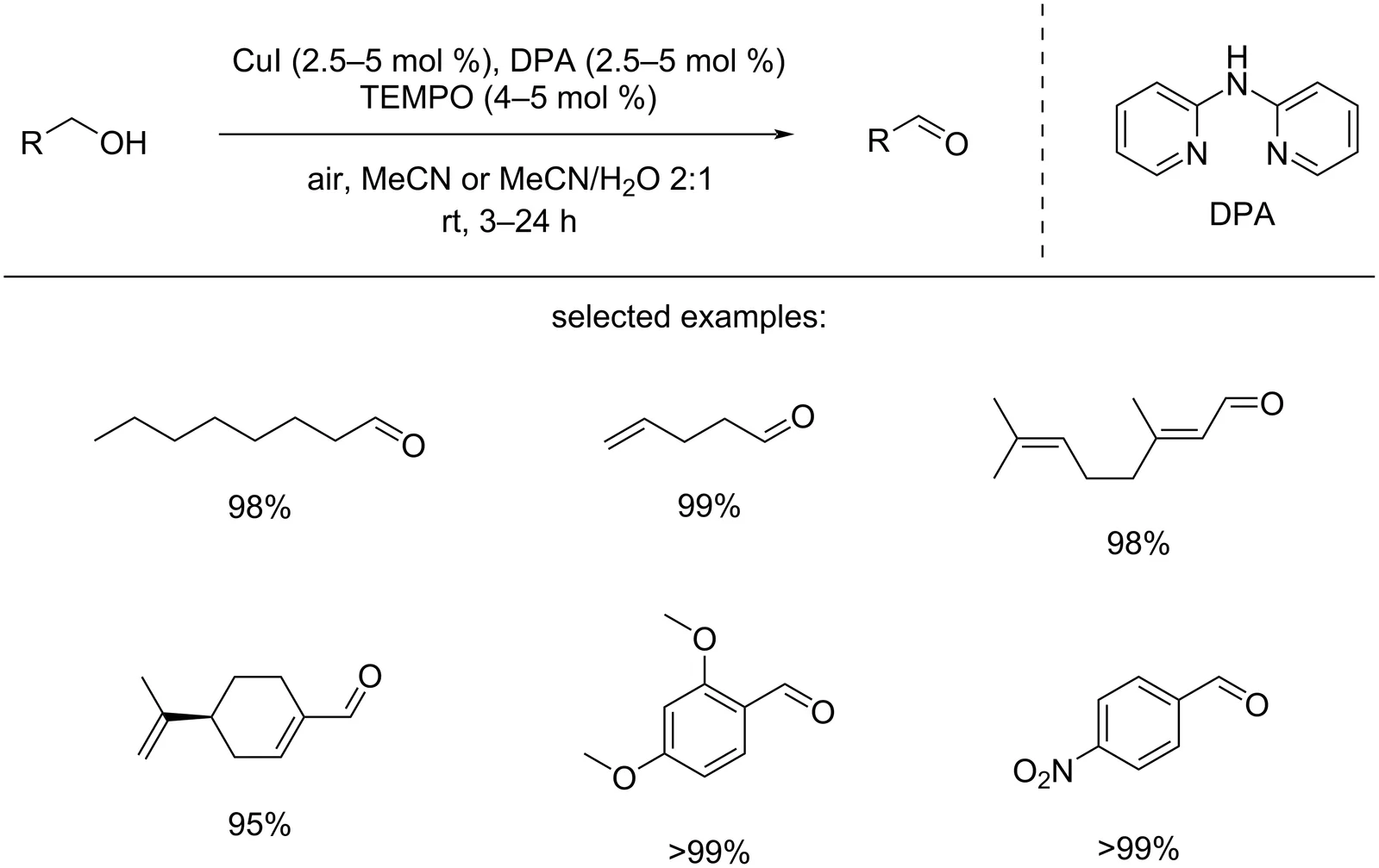
Cu/DPA/TEMPO base-free system for the oxidation of primary alcohols.

In 2022, the group of Repo [70] reported a base-free system comprised of the pre-catalyst CuI and 2,2′-dipyridylamine (DPA) acting as a ligand, along with TEMPO, that oxidizes primary aliphatic and benzylic alcohols at room temperature under air with nearly quantitative yields (Scheme 12). The authors also demonstrated the stability and applicability of their catalytic system under aqueous conditions.

**Scheme 13:**
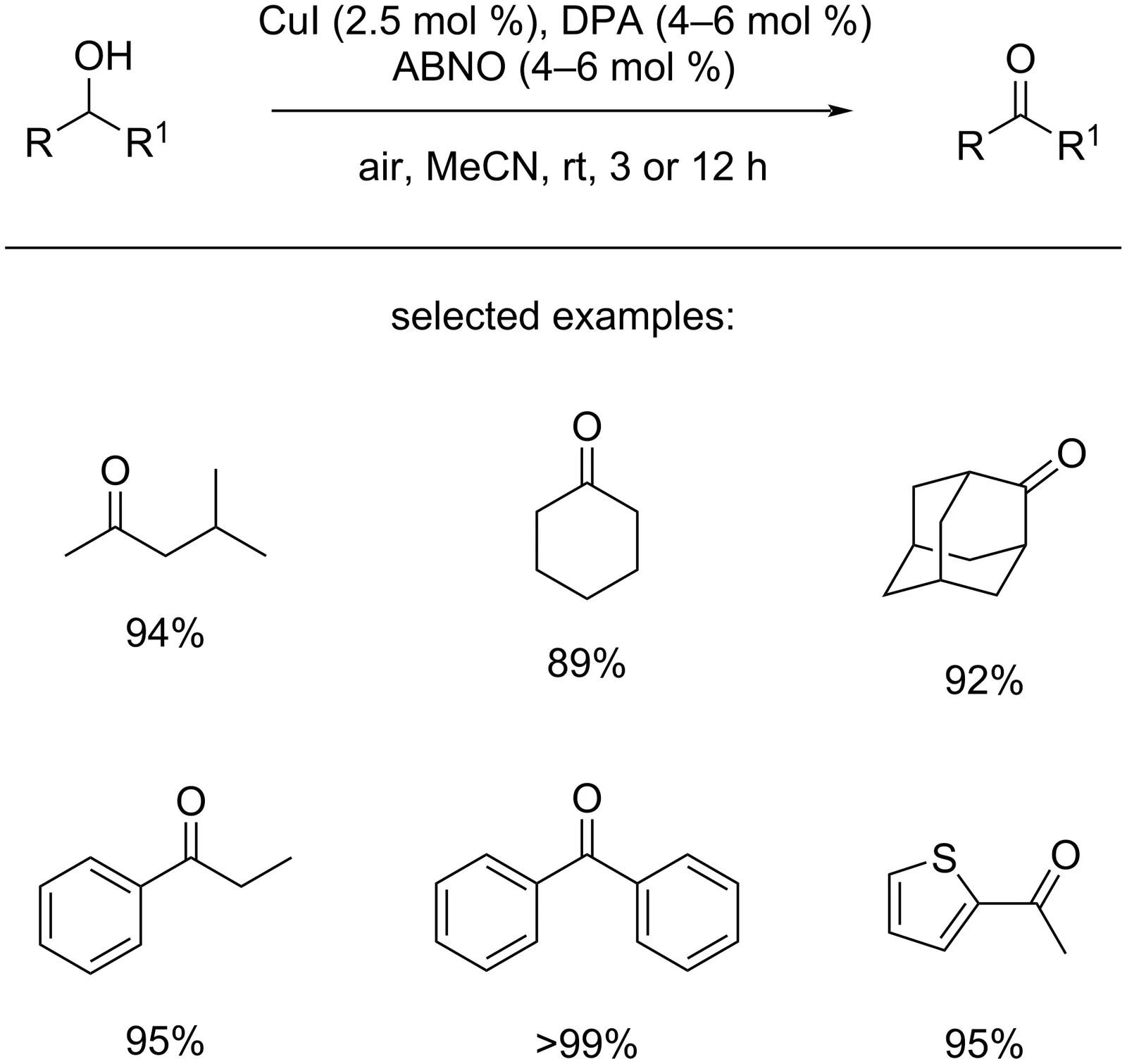
Cu/DPA/ABNO base-free system for the oxidation of secondary alcohols.

In 2025, the same group reported a modified version, adapted for the oxidation of secondary alcohols [71]. By substituting TEMPO with ABNO, the authors expanded the substrate scope while employing the same DPA ligand (Scheme 13).

**Scheme 14:**
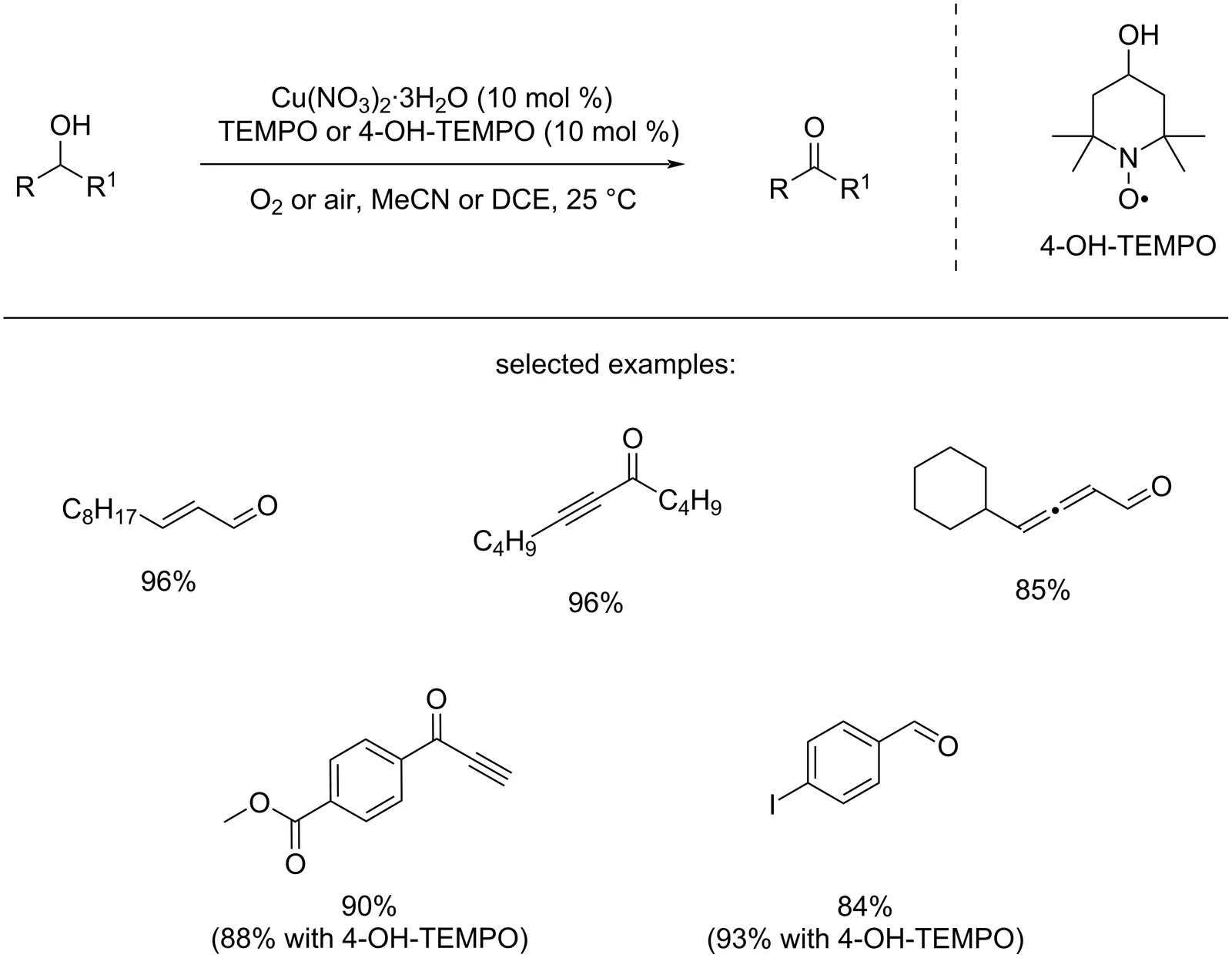
Base- and ligand-free Cu-catalyzed aerobic oxidation of alcohols.

Zhai and Ma demonstrated that a Cu-catalyzed base- and ligand-free aerobic method can successfully oxidize a wide scope of allylic, propargylic, and benzylic alcohols, as well as allenols [72]. The process utilizes the cheap Cu(NO_3_)_2_·3H_2_O salt coupled with TEMPO. Moreover, the authors showed the possibility of substituting TEMPO with the much cheaper 4-hydroxy-TEMPO (4-OH-TEMPO) while maintaining comparable outcome (Scheme 14).

#### Nitroxyl radical-free Cu-catalyzed aerobic alcohol oxidation

Early reports on nitroxyl radical-free Cu-mediated aerobic alcohol oxidation reactions focused on simple homogeneous complexes with nitrogenous ligands, such as pyridine, 2,2′-bipyridine, and 1,10-phenanthroline. These organic ligands primarily function as electron donors that stabilize various metal oxidation states while creating a steric environment at the metal center and thereby enhancing catalytic activity and selectivity. However, these early methods suffered from limited substrate scope since only activated benzylic and allylic alcohols could provide the corresponding aldehydes or ketones with satisfactory efficiency, whereas aliphatic alcohols proved incompatible. In several instances they were unreactive or underwent side reactions such as competing C–C-bond cleavage or over-oxidation to carboxylic acids [73–76]. Despite the common drawbacks, these preceding works have paved the way for more in-depth research in the field and later catalytic aerobic oxidations based on copper that do not rely on nitroxyl radicals have emerged. These protocols have brought improvements in terms of substrate scope, selectivity, and conversion.

**Scheme 15:**
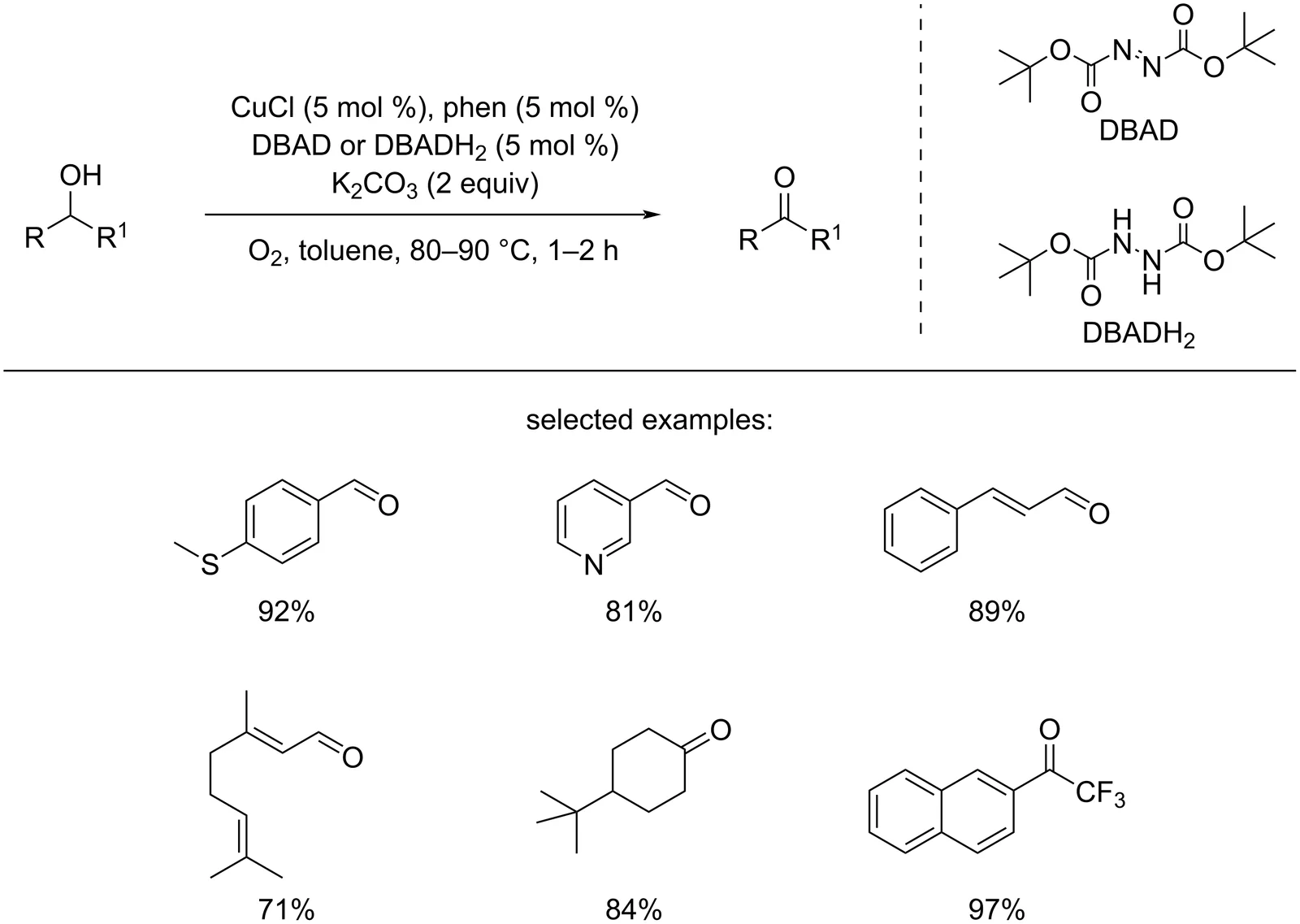
Oxidation of alcohols catalyzed by Cu/phen/DBADH_2_.

Markό and co-workers pioneered much of the copper-catalyst development between the end of the 20th and the beginning of the 21st century. In their initial report in 1996, a combination of CuCl, 1,10-phenanthroline, and di-*tert*-butyl azodicarboxylate (DBAD) or di-*tert*-butyl hydrazodicarboxylate (DBADH_2_), allowed the oxidation of alcohols with great functional group tolerance [77]. The method was applicable to a wide range of primary, secondary, allylic, and benzylic alcohols which were smoothly oxidized to the corresponding aldehydes or ketones with yields ranging from 71 to 97% (Scheme 15).

**Scheme 16:**
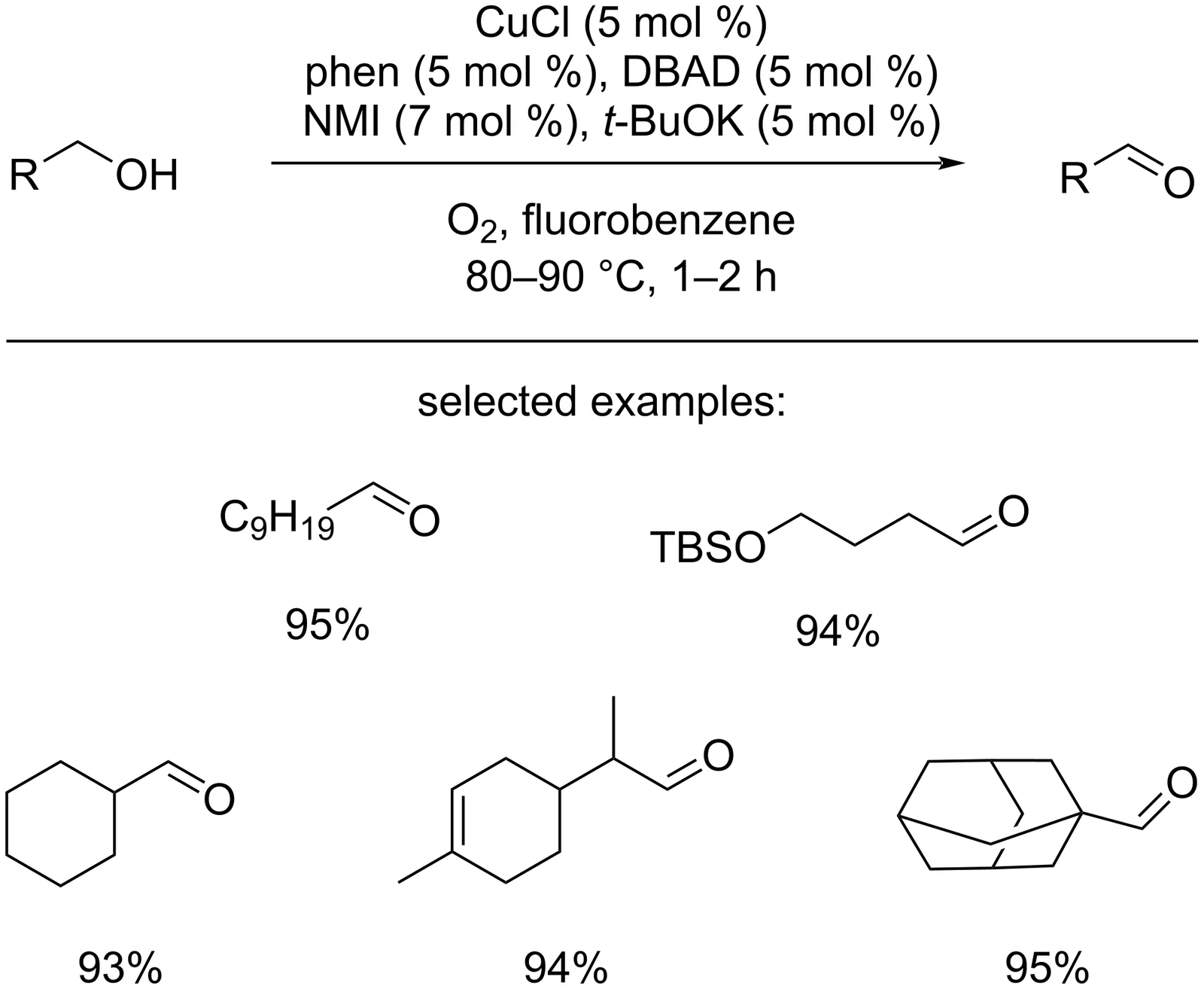
Improved protocol for primary aliphatic alcohol oxidation by Markό et al. [78].

However, this system required the presence of 2 equivalents of a base (K_2_CO_3_) and was not consistent for the oxidation of primary aliphatic alcohols. To address this limitation, the same group [78] later improved the previously established protocol. They have found that a change of the solvent from toluene to fluorobenzene allowed the use of a catalytic amount of base (*t*-BuOK in 5 mol %). The addition of *N*-methylimidazole (7 mol %) further boosted the efficiency of the system ensuring high conversion of various primary aliphatic alcohols (Scheme 16).

**Scheme 17:**
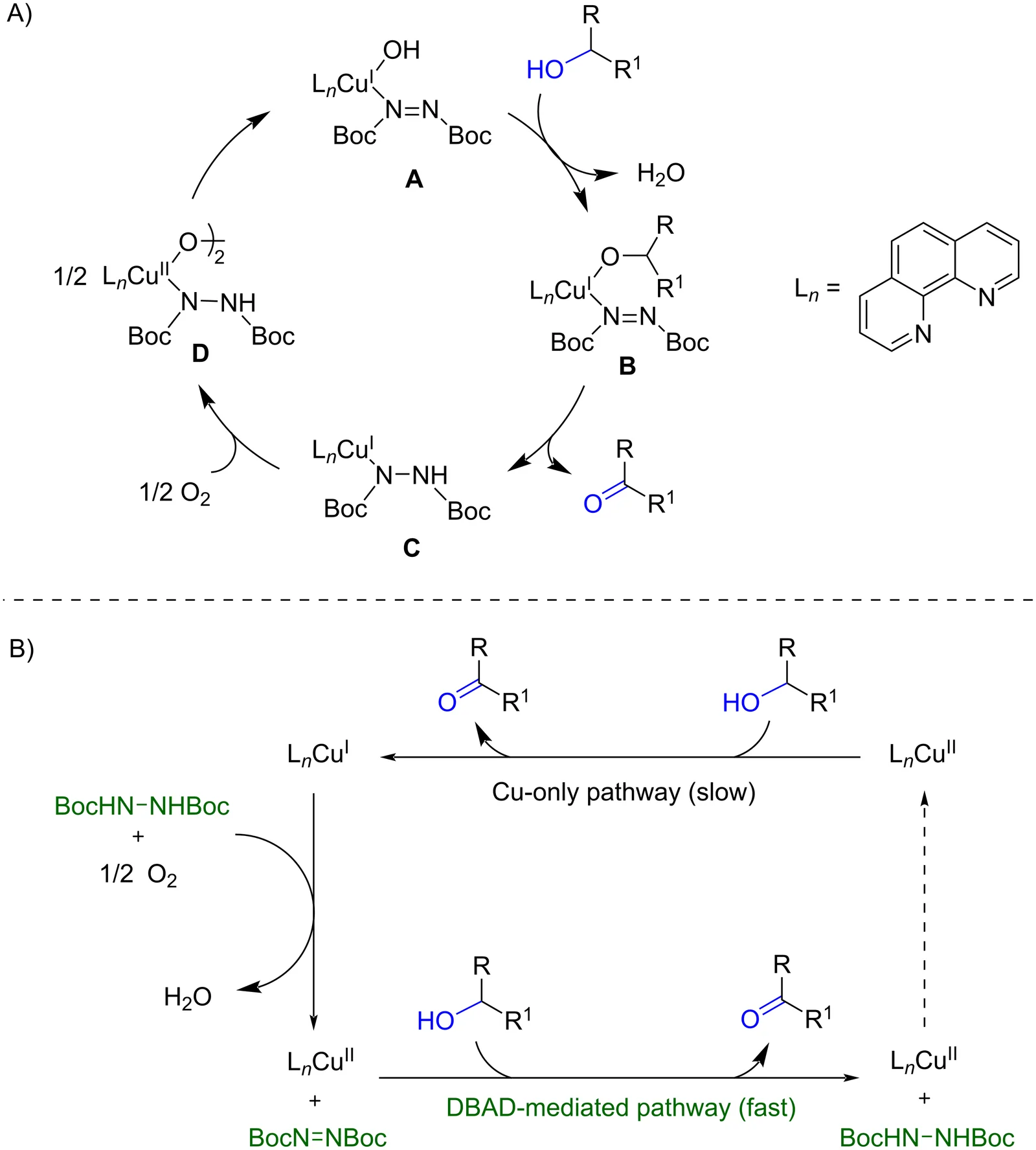
Mechanism of the Cu/DBAD-catalyzed oxidation: A) initial proposal by Markό et al.; B) further elucidation by McCann and Stahl.

The mechanism of the reaction proposed by Markό et al. is shown in Scheme 17A. The catalytic cycle initiates via a ligand exchange between Cu(I) phenanthroline hydroxide complex **A** and the alcohol substrate to yield the corresponding alkoxy species **B**. Subsequent alcohol oxidation to the target carbonyl compound proceeds concomitantly with diazodicarboxylate reduction to a hydrazide complex, followed by aldehyde dissociation that yields Cu(I) complex **C**. Aerobic oxidation of **C** then generates Cu(II) μ-peroxo species **D**. Finally, a homolytic peroxide bond (O–O) cleavage, which occurs with reduction of the copper center and oxidation of the hydrazide, restores the active Cu(I) catalyst **A** (Scheme 17A) [29,77,79]. Almost 20 years later, McCann and Stahl [80] conducted a detailed mechanistic study and further elucidated the mechanism which was initially proposed by Markό et al. [79]. They revealed that the reaction is driven by two interconnected catalytic cycles operating in succession. It begins with a rapid Cu/DBAD-mediated step where DBAD acts as the oxidant, followed by a slower, rate-limiting Cu-only pathway (Scheme 17B).

**Scheme 18:**
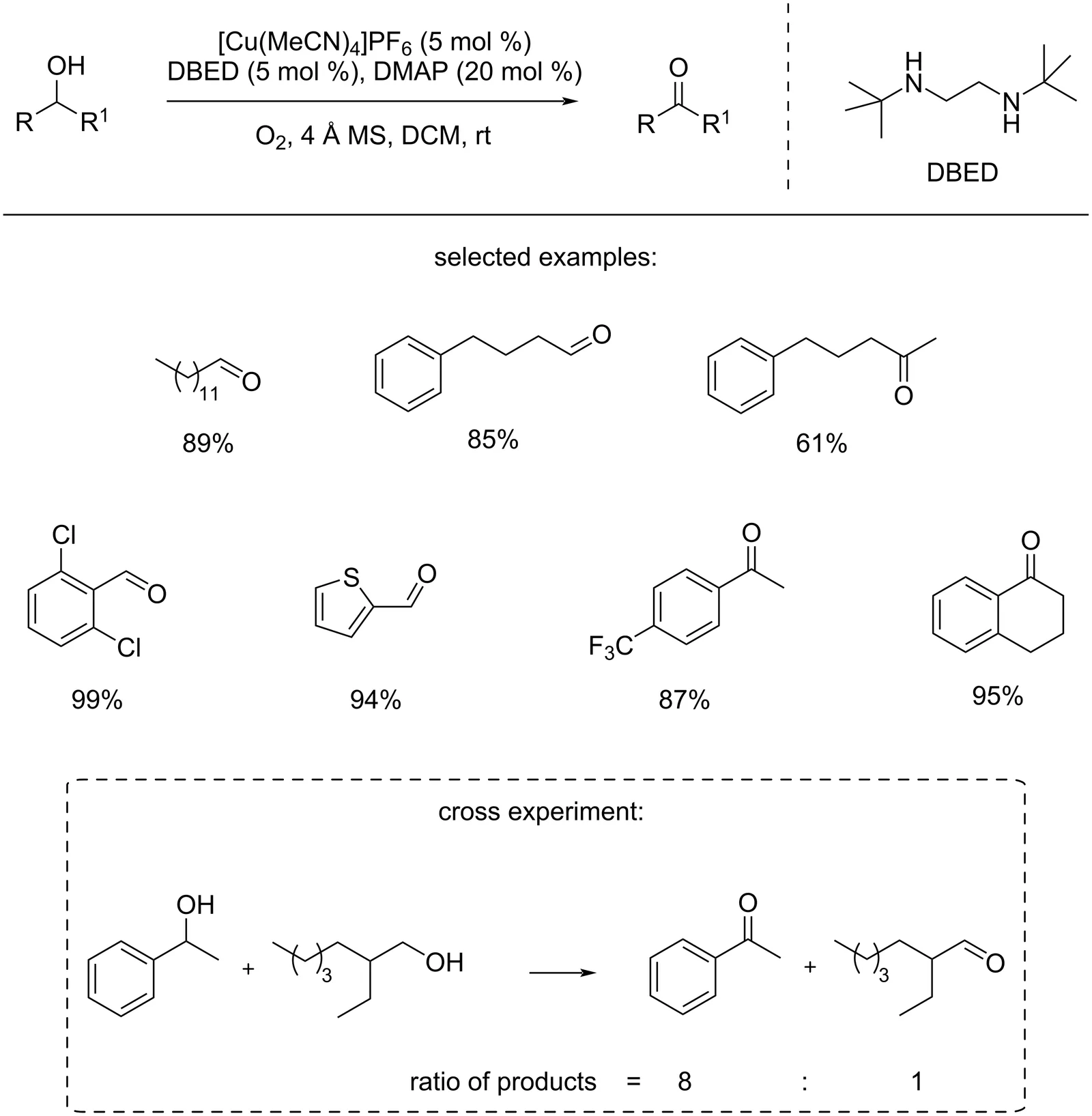
TEMPO-free Cu-catalyzed aerobic oxidation of alcohols reported by Arndtsen and Lumb [81].

A method, initially described as nitroxyl radical-free, for Cu-catalyzed aerobic oxidation of alcohols was published by the groups of Arndtsen and Lumb in 2015 [81]. The approach utilizes [Cu(MeCN)_4_]PF_6_, *N*,*N’*-di-*tert*-butylethylenediamine (DBED) as a ligand and 4-dimethylaminopyridine (DMAP) as a base. A variety of activated as well as non-activated substrates were well tolerated. The authors proved that the developed Cu/DBED-catalyzed oxidation offers important complementary selectivity to TEMPO-based systems, and favors the oxidation of activated secondary over primary alcohols (Scheme 18).

**Scheme 19:**
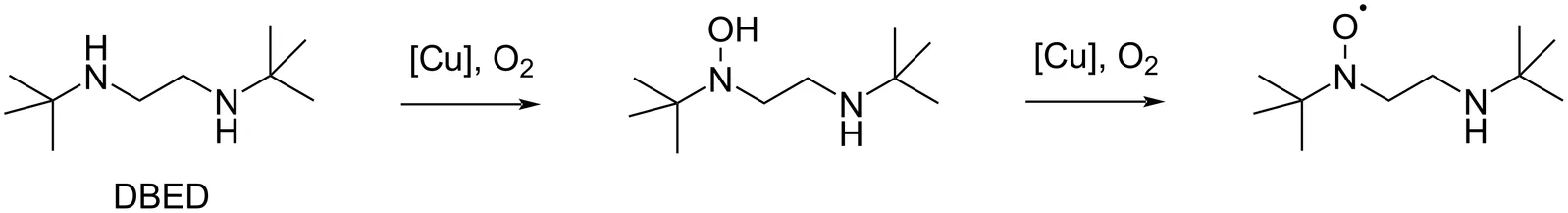
In situ generation of *N*-oxyl radicals from DBED.

Two years later, however, a mechanistic study performed by the groups of Arndtsen and Stahl [82] challenged the initial claim that the method proceeds in the absence of nitroxyl radicals. The authors proved such species can, in fact, be generated in situ under the reaction conditions. Influenced by the Cu catalyst and oxygen atmosphere, the ligand, DBED, undergoes N-hydroxylation leading to the formation of a nitroxyl-type species (Scheme 19) which subsequently acts as a hydrogen atom abstractor – a role analogous to that of TEMPO in related oxidation systems.

**Scheme 20:**
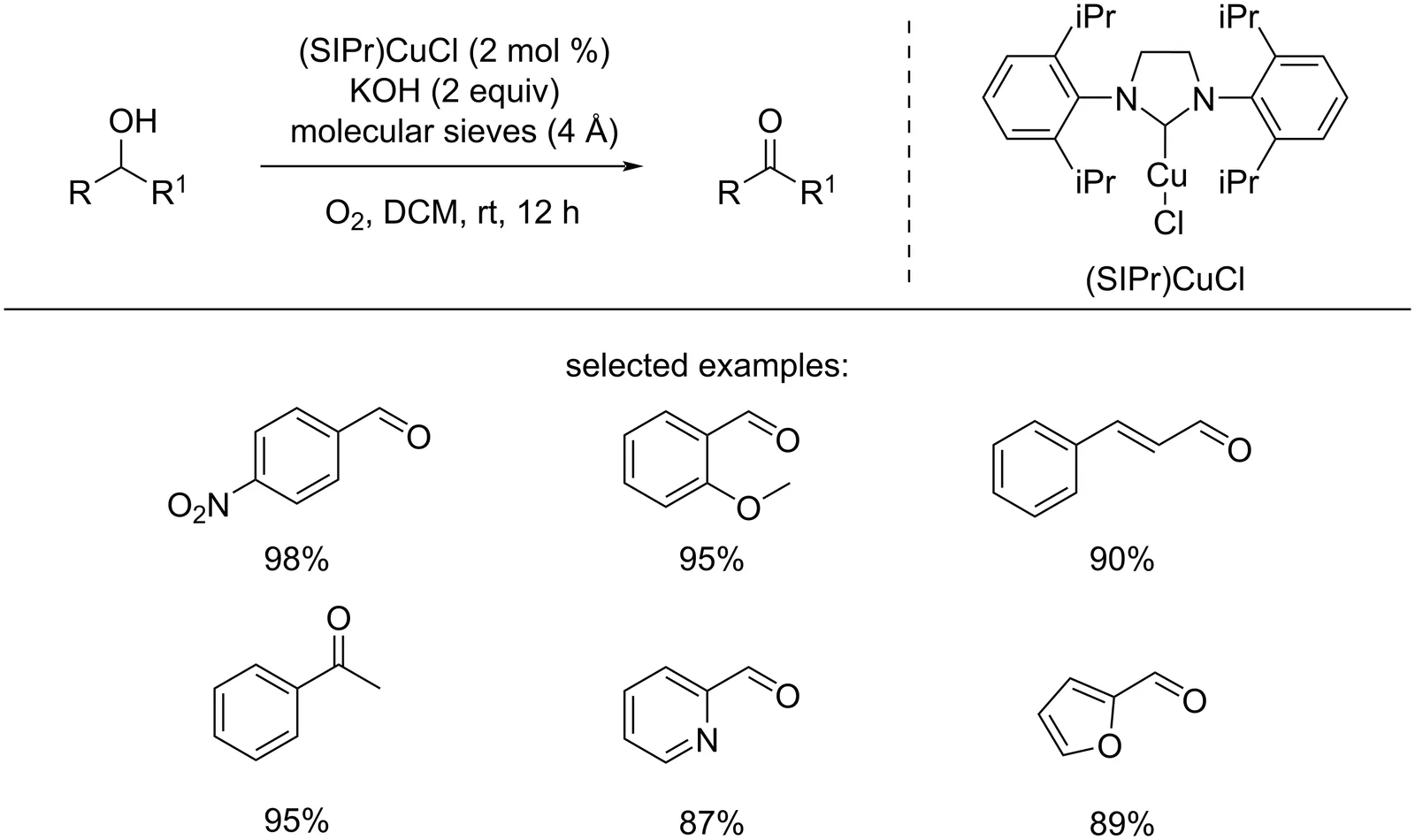
(SIPr)CuCl-catalyzed aerobic oxidation of alcohols.

Jiang et al. reported a mild Cu/NHC-catalyzed (NHC = N-heterocyclic carbene) aerobic oxidation of benzylic and heteroaromatic alcohols [83]. The method utilizes the commercially available NHC complex (SIPr)CuCl. In the presence of molecular sieves both primary and secondary alcohols were converted to the corresponding carbonyl products with high isolated yields (Scheme 20). However, beyond the requirement of two equivalents of a base (KOH), a major drawback of this protocol is its failure to oxidize aliphatic substrates. Despite these disadvantages, the authors highlighted the practicality of the protocol and achieved the synthesis of valuable imines and amines in a cascade one-pot fashion.

**Scheme 21:**
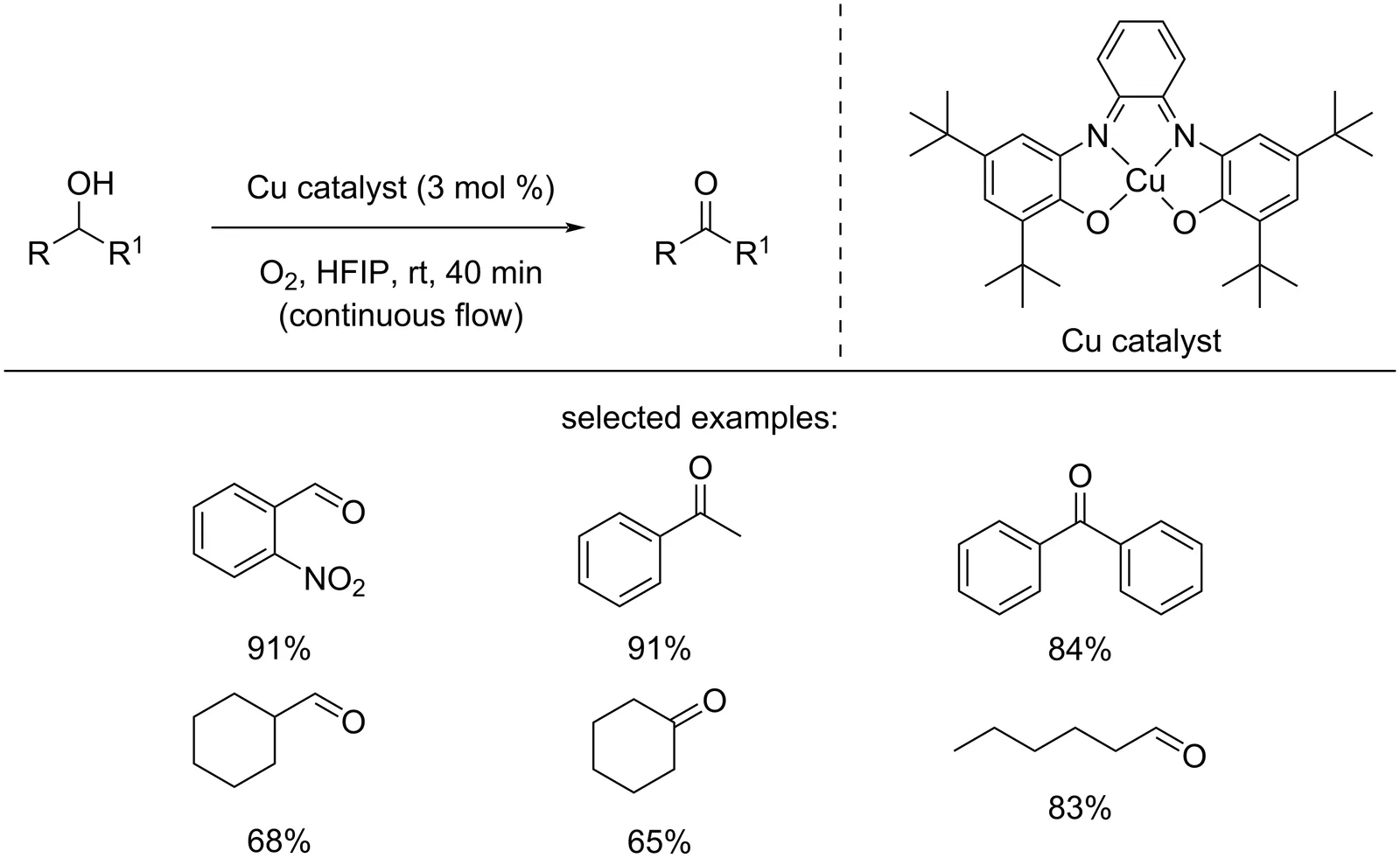
Base- and nitroxyl radical-free Cu-catalyzed aerobic oxidation of alcohols under continuous-flow conditions.

Z. Zhang and F. Zhang et al. introduced another nitroxyl radical-free approach for alcohol oxidation [84]. Their method implements a Cu catalyst featuring a redox-active ligand under an atmosphere of oxygen, without the need for a base additive. As shown in Scheme 21, a range of primary, secondary, aromatic, and aliphatic alcohols were smoothly oxidized at ambient temperature in hexafluoroisopropanol (HFIP). The Cu catalyst, however, is not an off-the-shelf material and must be prepared ex situ via a two-step procedure prior to the catalytic reaction. Despite this drawback, the authors successfully adapted the protocol to continuous-flow conditions, which significantly enhanced the reaction rate and reduced the reaction duration from 24 hours (for the batch conditions) to just 40 min.

The utility and scalability of Cu-catalyzed aerobic oxidations have also attracted interest for pilot- and industrial-scale applications. Demonstrations of such protocols include process-intensified setups [85–86], as well as patented upscale methodologies for oxidations of primary and secondary alcohols [87–88]. Furthermore, the value of copper-catalyzed aerobic oxidation is increasingly amplified by the growing availability of biosourced alcohols derived from renewable biomass. Converting bio-derived chemicals such as 5-hydroxymethylfurfural [89–91] and various α-ketoalcohols [92–93] into high-value products under mild Cu-catalyzed conditions represents a key pillar of sustainable organic synthesis.

### Copper-catalyzed aerobic oxidation of aldehydes to carboxylic acids

**Scheme 22:**
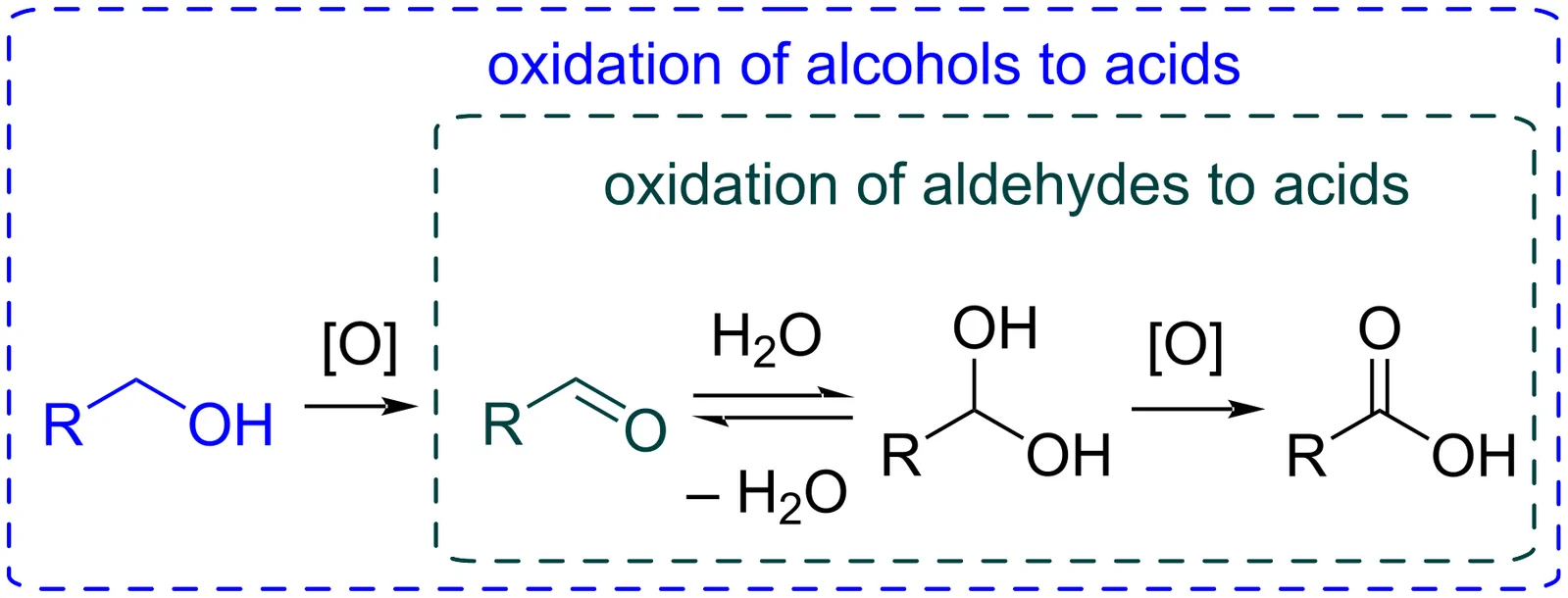
Carboxylic acids as products of aldehyde and alcohol oxidations – general mechanism.

Copper-catalyzed aerobic oxidation has been further developed to promote not only the oxidation of alcohols to aldehydes but also the conversion of the latter into the corresponding carboxylic acids. While many catalytic systems are designed to achieve selective oxidation of alcohols and typically stop at the aldehyde stage, a number of methodologies have been reported that enable complete oxidation. The vast majority of existing protocols require the presence of water in the reaction mixture to establish an equilibrium between the aldehyde and its corresponding hydrate (*gem*-diol), as the latter serves as the actual intermediate undergoing oxidation to the desired acid. In such systems, aldehydes can be efficiently transformed into the corresponding carboxylic acids, and in certain cases, alcohols can be directly converted into acids in a one-pot fashion (Scheme 22) [94], which is particularly attractive from both the synthetic and the green chemistry standpoint.

Over the past decades, significant efforts have been directed toward the development of transition-metal-catalyzed aerobic oxidation of aldehydes to carboxylic acids, with early methodologies largely based on noble metal catalysts, e.g., palladium (Pd) [95], platinum (Pt) [95], gold (Au) [96], silver (Ag) [97], etc. Nonetheless, the cost and scarcity of precious metals limit their practicality. Therefore, exploring earth-abundant metals, such as Cu, is of significant importance.

**Scheme 23:**
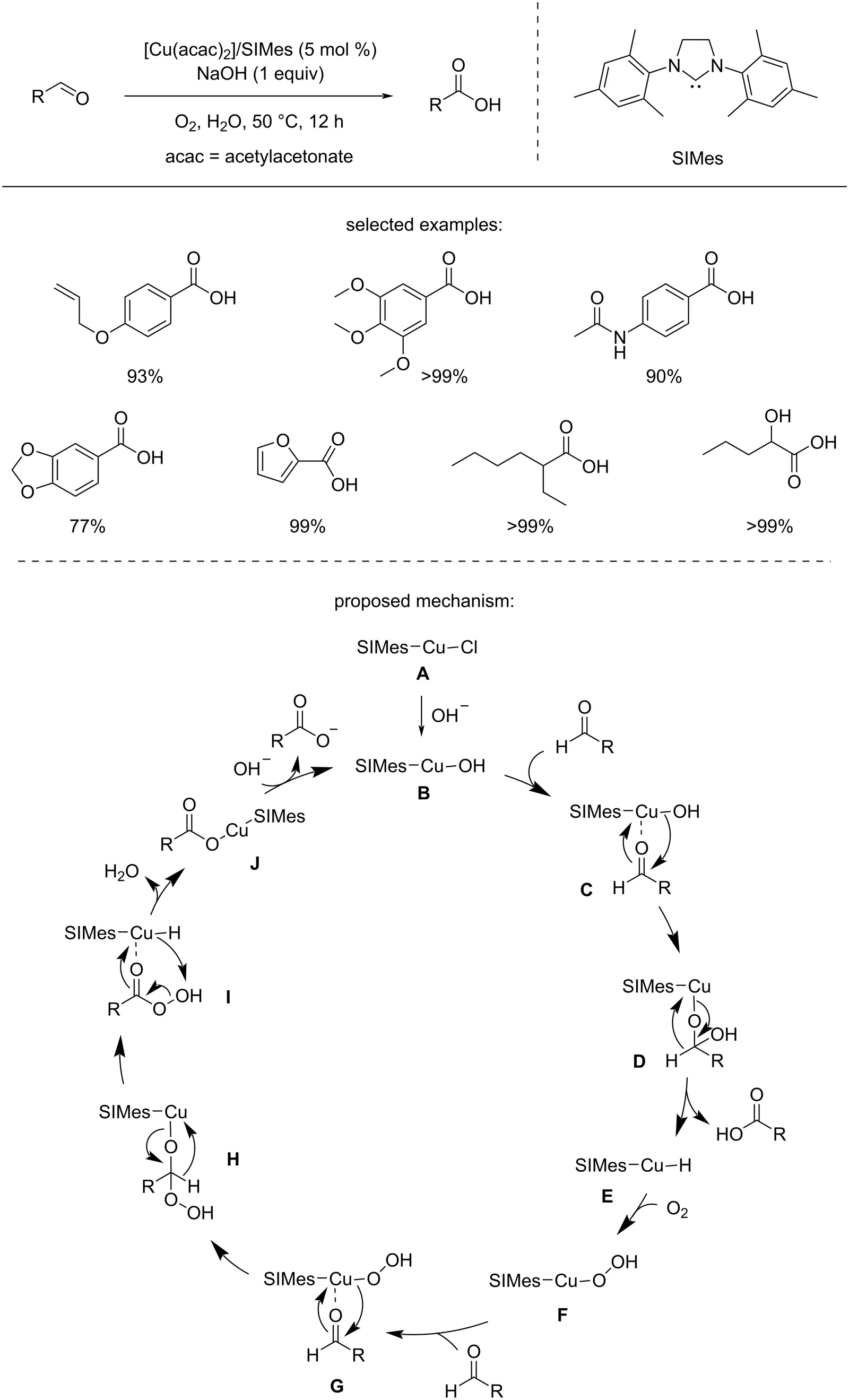
The first homogeneous Cu-catalyzed aerobic oxidation of aldehydes.

In 2016, the first example of homogeneous copper-catalyzed aerobic oxidation of aldehydes was presented [98]. The method utilizes a Cu/NHC system, namely [Cu(acac)_2_]/SIMesCuCl, under atmospheric oxygen. The reported reaction proceeds under mild aqueous conditions, and covers a wide range of functionalized aldehydes (Scheme 23). Another advantage of this approach is that chromatography is generally not necessary for product purification. However, stoichiometric amount of a base (NaOH) is required. Identified by the authors as a catalytic Fehling’s reaction, the proposed mechanism (depicted in Scheme 23) begins with the hydroxide-mediated conversion of NHC complex SIMesCuCl (**A**) into the active SIMesCuOH (**B**). Aldehyde coordination to **B** generates **C**, which is attacked by a hydroxide ion to yield intermediate **D**. Subsequent β-hydride elimination affords the hydride **E**. Reaction of **E** with molecular oxygen affords hydroperoxide **F**, which coordinates a second aldehyde to form **G**. Attack of the hydroperoxide in **G** produces intermediate **H**, which undergoes hydride abstraction to form **I**. An intramolecular hydride-mediated peroxide reduction within **I** furnishes the carboxylate complex **J**. The catalytic cycle closes upon hydroxide substitution of the carboxylate, releasing the product and regenerating **B** [98].

**Scheme 24:**
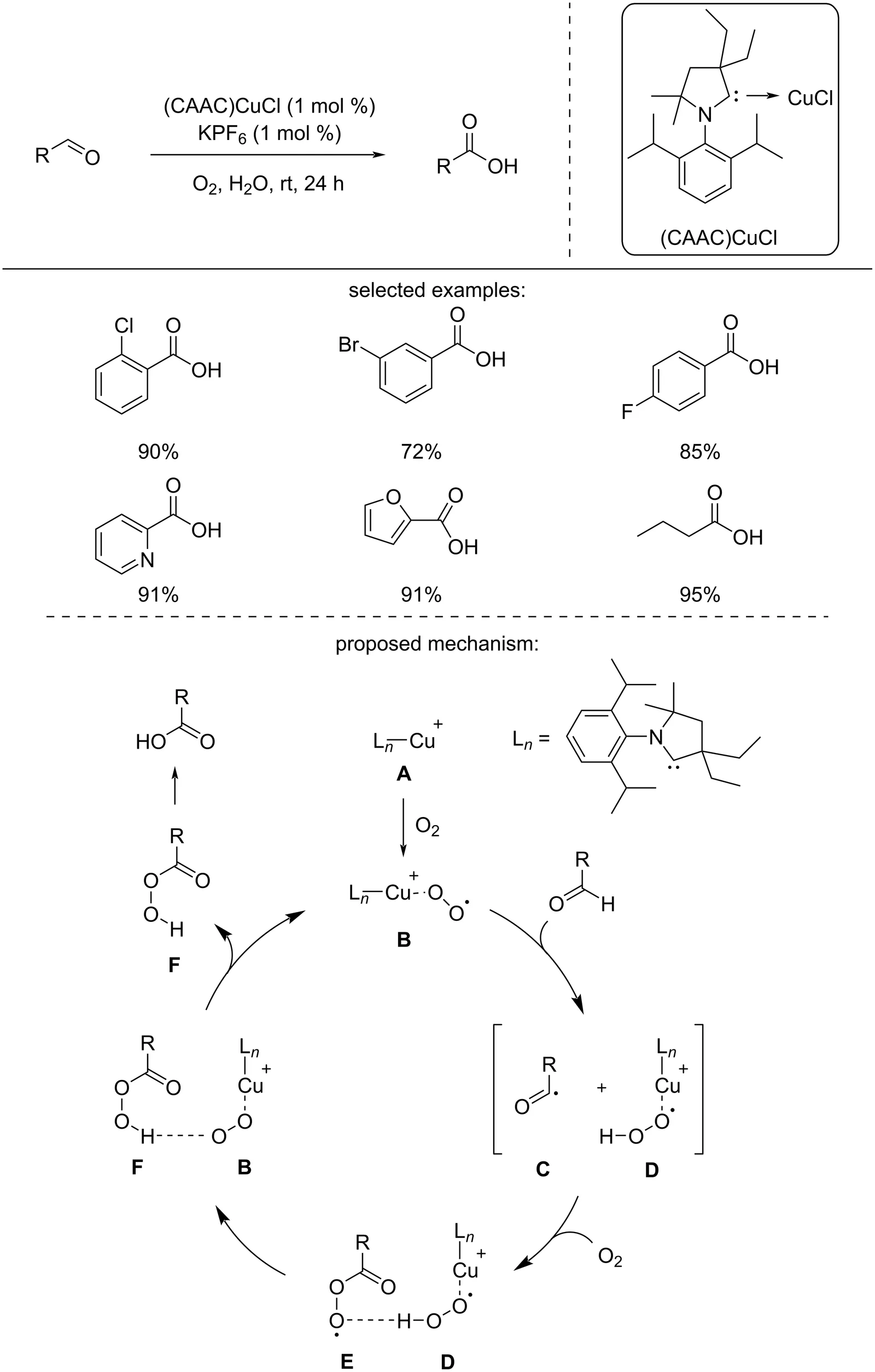
(CAAC)CuCl-catalyzed aerobic oxidation of aldehydes.

In 2022, a similar to the NHC-based copper catalysts, namely cyclic (alkyl)(amino)carbene copper chloride ((CAAC)CuCl) was reported to promote the aerobic oxidation of aldehydes (Scheme 24) [99]. The method operates at room temperature in water and uses only a catalytic amount of an additive (1 mol % of KPF_6_) which is necessary for the release of the active cation **A**. The authors’ mechanistic proposal is shown in Scheme 24. Upon generation of **A**, activation of molecular oxygen produces species **B**, which then abstracts the aldehyde’s formyl hydrogen atom to form radical species **C** and **D**. In the presence of O_2_, an acyl peroxy radical **E** is generated, which in turn re-abstracts the hydrogen of species **D** yielding peroxy acid **F**, and the cycle closes with the regeneration of species **B**. Due to the unstable nature of **F** under aqueous conditions, the final carboxylic acid product is formed [99].

The above-mentioned methods, however, share a major drawback – the catalyst (a complex between a copper salt and a carbene ligand) must be prepared ex situ in advance, which complicates the overall synthetic procedure by requiring additional preparation steps prior to the catalytic reaction.

**Scheme 25:**
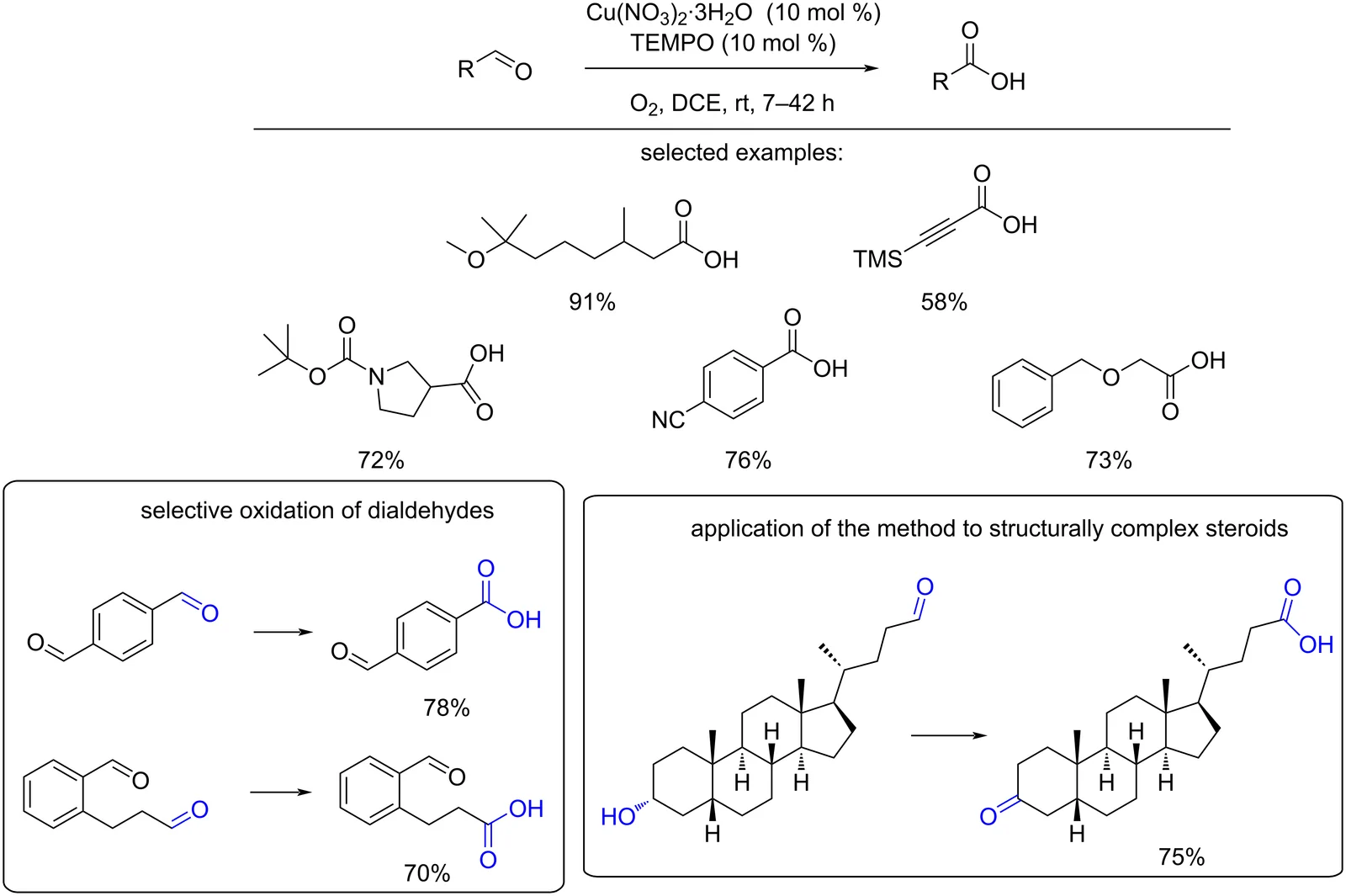
Cu(NO_3_)_2_·3H_2_O/TEMPO-catalyzed selective oxidation of aldehydes.

The most recent report on aerobic Cu-catalyzed oxidation of aldehydes, published in 2025 by the group of Ma, overcomes these disadvantages by offering an operationally simple ligand-free procedure that utilizes a cheap and readily available salt, namely Cu(NO_3_)_2_·3H_2_O, in combination with TEMPO as a co-catalyst [100]. The transformation proceeds under mild conditions in 1,2-dichloroethane (DCE) and tolerates an array of functional groups. Moreover, the approach was proved selective towards different aldehyde functionalities, and its utility was showcased in the transformation of structurally complex steroid molecules (Scheme 25).

**Scheme 26:**
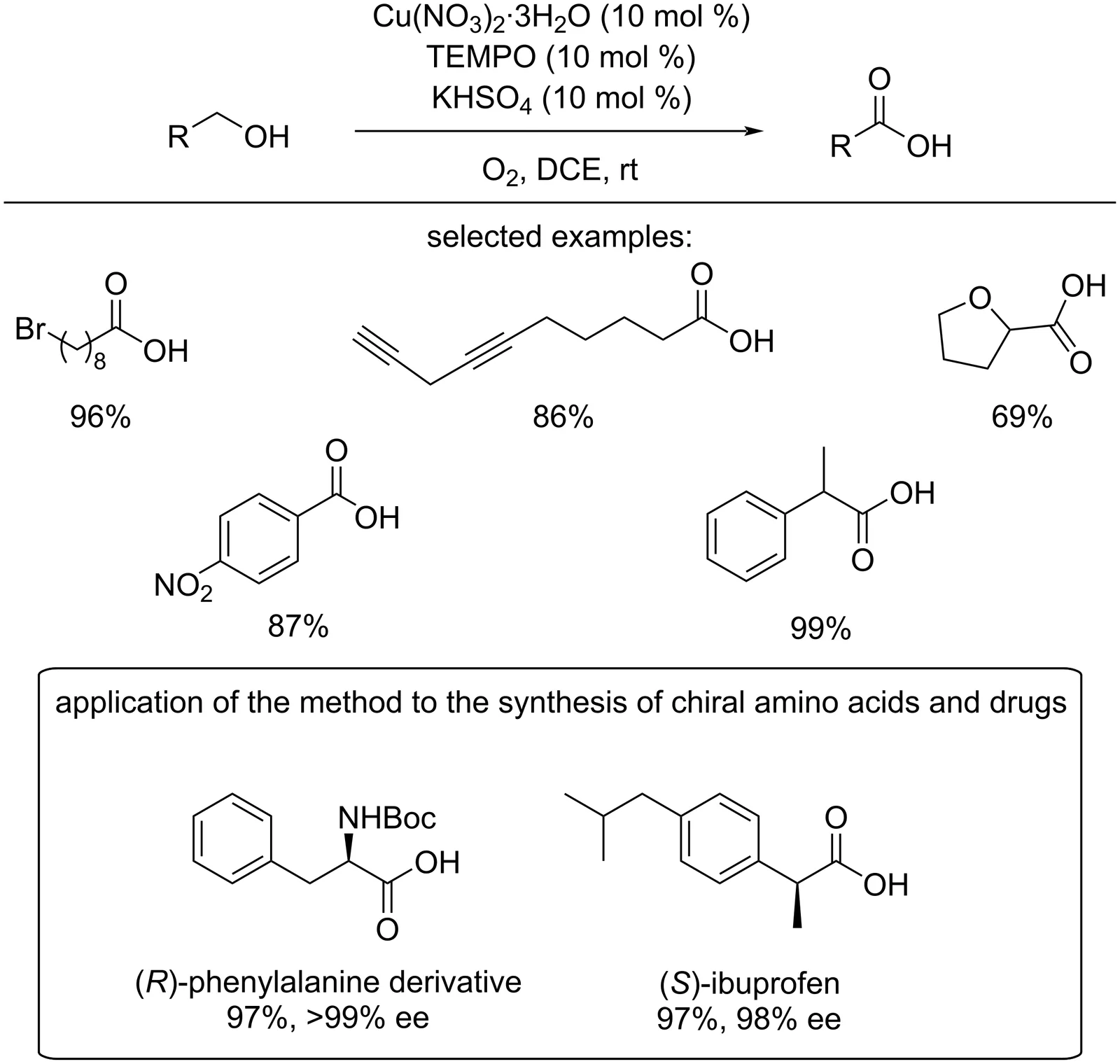
Cu(NO_3_)_2_·3H_2_O/TEMPO/KHSO_4_-catalyzed direct oxidation of alcohols to carboxylic acids.

The group of Ma also reported an approach in which primary alcohols were efficiently and directly oxidized to acids employing catalytic amounts of Cu(NO_3_)_2_·3H_2_O, TEMPO, and KHSO_4_ under O_2_ [101]. Alcohols containing aliphatic, benzylic, alkynyl, amino, as well as sterically demanding groups were readily oxidized in high isolated yields. In addition, starting from chiral alcohol precursors the synthesis of important amino acids and drugs was achieved without racemization (Scheme 26).

**Scheme 27:**
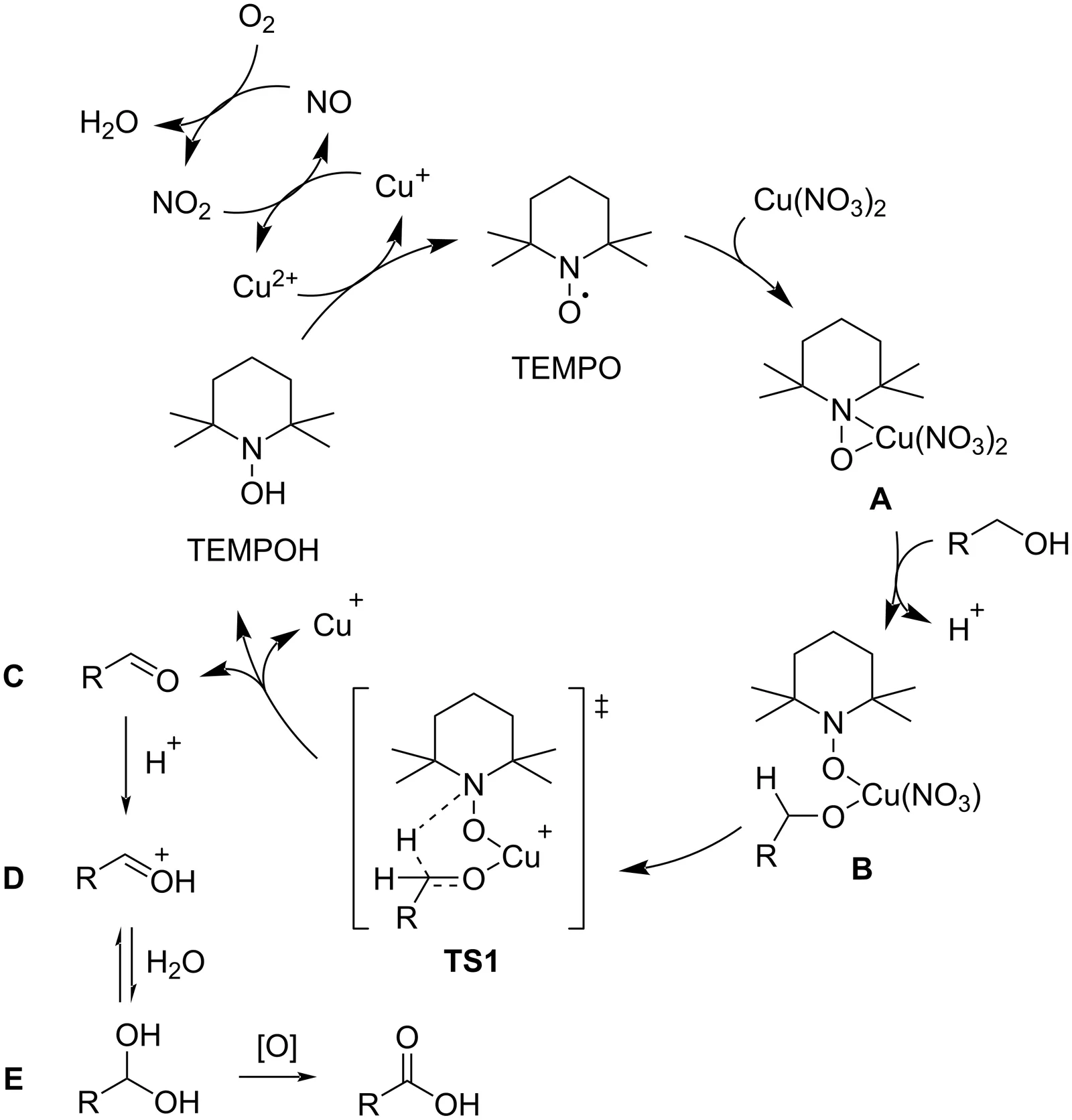
Proposed mechanism for the Cu(NO_3_)_2_·3H_2_O/TEMPO/KHSO_4_-catalyzed direct conversion of alcohols to acids.

A mechanistic study showed that both bisulfate (KHSO_4_) and water are essential for the reaction. The former facilitates the alcohol oxidation, whereas water ensures the hydration of the intermediate aldehyde, consequently accelerating the overall reaction [102]. The mechanism proposed by the group of Ma for the direct transformation of primary alcohols into acids is depicted in Scheme 27. First, intermediate **A** is formed from TEMPO and Cu(NO_3_)_2_, which then undergoes a ligand exchange with the alcohol substrate to generate **B**. Hydrogen-atom abstraction through the cyclic transition state **TS1** yields TEMPOH, Cu^+^, and aldehyde **C**. Oxidation of TEMPOH by Cu^2+^ regenerates TEMPO, whereas Cu^2+^ is restored by oxidation of Cu^+^ with NO_2_. Protonation of aldehyde **C** affords intermediate **D**, which is then hydrated to **E**. Finally, oxidation of *gem*-diol **E** via the same catalytic pathway as for the alcohol furnishes the carboxylic acid product [102].

## Summary and Outlook

Copper-catalyzed aerobic oxidation of alcohols and aldehydes represents a highly valuable transformation in modern synthetic chemistry. A defining advantage of these reactions is the use of molecular oxygen as the terminal oxidant, offering excellent atom economy, low cost, and reduced environmental impact compared to traditional stoichiometric oxidants.

The success of these methodologies is closely linked to the accessibility and versatility of copper. Copper, as an earth-abundant transition metal, provides a sustainable alternative to noble metals while maintaining rich and tunable redox behavior. Its ability to operate within diverse catalytic systems, including those involving organic co-catalysts or ligand-assisted pathways, underpins the broad applicability of aerobic oxidation chemistry.

In the context of green chemistry, copper-catalyzed aerobic oxidation aligns well with key principles such as the use of benign oxidants, waste minimization, and the avoidance of toxic or precious metals, particularly when implemented under operationally simple conditions.

Despite the considerable progress achieved in this field, important challenges remain. Many of the reported systems rely on the presence of additional components such as nitroxyl mediators, tailored ligands, or basic additives, which can complicate reaction design and limit practical applicability. Recent advances, however, suggest that simpler catalytic systems, potentially operating in the absence of external mediators, ligands, and/or base additives, are within reach. These emerging examples underscore the need for continued efforts in catalyst discovery, systematic screening, and mechanistic investigation within the synthetic community.

Furthermore, the development of methodologies based on readily available, off-the-shelf reagents and catalysts should be considered a priority. In many practical settings, such systems are strongly preferred over in-house synthesized complexes, as they enhance the operational simplicity, reproducibility, and broader accessibility of the protocols. Progress along these lines is expected to further consolidate copper-catalyzed aerobic oxidation as a cornerstone transformation in sustainable organic synthesis.

## Data Availability

Data sharing is not applicable as no new data was generated or analyzed in this study.
